# A Survey on Reduction of Energy Consumption in Fog Networks—Communications and Computations

**DOI:** 10.3390/s24186064

**Published:** 2024-09-19

**Authors:** Bartosz Kopras, Filip Idzikowski, Hanna Bogucka

**Affiliations:** Faculty of Computing and Telecommunications, Poznan University of Technology, 60-965 Poznan, Poland; bartosz.kopras@doctorate.put.poznan.pl (B.K.); filip.idzikowski@put.poznan.pl (F.I.)

**Keywords:** survey, fog network, optimization, energy-efficiency, edge computing

## Abstract

Fog networking has become an established architecture addressing various applications with strict latency, jitter, and bandwidth constraints. Fog Nodes (FNs) allow for flexible and effective computation offloading and content distribution. However, the transmission of computational tasks, the processing of these tasks, and finally sending the results back still incur energy costs. We survey the literature on fog computing, focusing on energy consumption. We take a holistic approach and look at energy consumed by devices located in all network tiers from the things tier through the fog tier to the cloud tier, including communication links between the tiers. Furthermore, fog network modeling is analyzed with particular emphasis on application scenarios and the energy consumed for communication and computation. We perform a detailed analysis of model parameterization, which is crucial for the results presented in the surveyed works. Finally, we survey energy-saving methods, putting them into different classification systems and considering the results presented in the surveyed works. Based on our analysis, we present a classification and comparison of the fog algorithmic models, where energy is spent on communication and computation, and where delay is incurred. We also classify the scenarios examined by the surveyed works with respect to the assumed parameters. Moreover, we systematize methods used to save energy in a fog network. These methods are compared with respect to their scenarios, objectives, constraints, and decision variables. Finally, we discuss future trends in fog networking and how related technologies and economics shall trade their increasing development with energy consumption.

## 1. Introduction

### 1.1. Motivation

The Internet of Things (IoT) is rapidly expanding as the number of connected devices continues to grow [1,2]. The “things” often have small physical dimensions, little computational power, and low available storage. Big, efficient cloud Data Centers (DCs) are suited for storing and processing these data, especially if results are collected from many devices distributed over a large area. Some IoT applications require low latency. Mobile Cloud Computing (MCC) speeds up computations by offloading tasks from Mobile Devices (MDs) to powerful cloud servers [3,4]. However, MCC has limitations, so some authors propose cloudlets [5,6], i.e., smaller, distributed versions of cloud DCs.

Cisco followed up on the idea of decentralizing cloud computing in 2012. They proposed a novel paradigm—fog computing [7]—which brings computational resources closer to the end user and improves Quality of Service (QoS) by reducing latency. This avoids the unnecessary transmission of data to the cloud (long routes in terms of Internet Protocol (IP) hops, long round trip times, and unpredictable jitter) and takes into account limited resources of end stations. It is worth noting that fog computing is meant to improve the performance of the cloud and not to supersede it [7]. There are more and more things requiring computational help [8,9]. However, there are also more and more routers, Access Points (APs), servers, etc., which can provide computational help, i.e., become Fog Nodes (FNs). Software Defined Networks (SDNs) and Network Function Virtualization (NFV) are the enabling technologies for it [8]. Fog typically has a hierarchical, three-tier network structure. MDs, sensors, and all kinds of devices associated with the IoT are located in the bottom “things” tier. They are usually small in size, limited by their processing power or battery levels. The middle tier consists of one or more FNs, each with computational capabilities. Finally, in the top tier, there is usually one cloud with the greatest computing and storage resources.

Fog computing is often called a “greener” alternative to the cloud [10], which is especially important as the Information and Communication Technology (ICT) sector (and particularly cloud DCs) is projected to consume even more energy in the coming decade [11,12]. Therefore, in this work, we examine the topic of energy consumption and efficiency in the context of fog and other distributed computing networks.

We identify, through the analysis of related works, that there is no detailed review of modeling of the networks and especially of the parameterization of these models. We also notice that many works on the optimization of fog networks provide multiple different problems, optimization methods, and simulated scenarios. Still, the surveys that review them do not address this issue in their analysis.

### 1.2. Contributions

We make the following contributions in our work:

- We provide a thorough examination of the published survey works through the lenses of energy consumption. Forty-two such works are listed in Table 1, and their contents are compared with ours.
- We provide a holistic perspective on the energy consumption of fog networks related to both computing and communication and include perspectives of both wireless and wired parts of the network.
- Our detailed study includes a survey of network models, their parameterization, and optimization methods and technologies used to reduce the energy consumption of fog networks.
- Our analysis of optimization in the fog network is comprehensive, extracting key information such as objective functions, constraints, and decision variables from analyzed works. We analyze each work presenting multiple optimization problems and solutions. Together, this allows readers to compare and contrast optimization methods between works and within them.
- We discuss the results presented in the surveyed works with a focus on energy reduction but taking into account other metrics such as delay. Baseline solutions are consistently listed.
- We point out future research directions based on the current trends observed in the surveyed works and on our own experience.

We are convinced that the above-mentioned contributions are novel with respect to the 42 survey papers listed in Table 1, which in itself cannot be found in other works.

### 1.3. Work Outline

**Figure 1:**
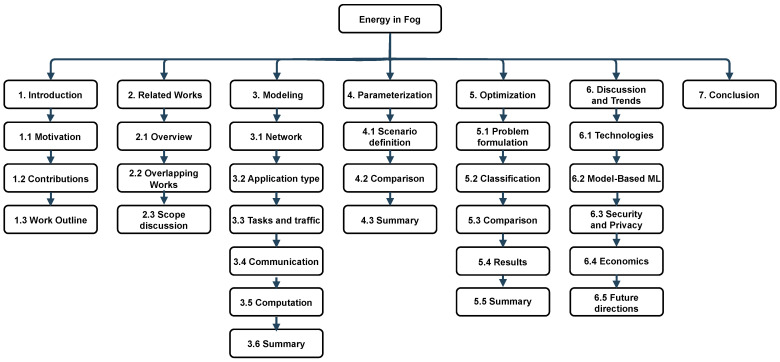
Work outline by section.

This work is structured as follows. We first provide an overview of previous related survey works, classifying them into various categories and showing the contributions of our work (Section 2). Next, in Section 3, we compare mathematical models describing fog: the network itself, tasks, traffic, and energy spent on computation as well as on communication. Then, we provide an analysis of the parameterization of these models in Section 4. In Section 5 we compare various works that focus on the optimization of the fog with various algorithms allocating tasks and resources. Later in Section 6, we discuss general trends based on the insights gathered from the surveyed papers and point out future research directions. Finally, Section 7 concludes our work. The acronyms used throughout the paper are listed in the Abbreviations section at the end of the paper. The composition of the work is shown in Figure 1.

## 2. Related Work

### 2.1. Overview of Published Surveys

Multiple survey works have been published on fog computing and related paradigms. A summary of their content is presented in Table 1 in the context of our work and its topics. ‘-’ in Table 1 means that the topic is not mentioned throughout the work. ‘∘’ means that the topic is briefly mentioned, without elaboration. Finally, ‘+’ means that the topic is examined to a certain extent. The columns of Table 1 refer to the focus of our work:

(1) *Energy consumption of fog—in General*—discussion or mentioning of energy consumed in the fog. Works that do not cover it such as [13,14] are not included in the Table 1.
(2) *Energy—Fog vs. Cloud*—comparing and contrasting the energy consumption of solutions based on fog (possibly including cloud) and those based solely on cloud.
(3) *Energy for Communication*—energy spent on the transmission of data between nodes in the network. We examine whether the works discuss/present mathematical models describing the energy consumption in the fog. We tick the *General* column if the topic is at least briefly tackled. The *Model* column is ticked if network models (including traffic, nodes, and communications) are discussed in the survey, while the *Param*eterization column is ticked if the model parameter values are surveyed.
(4) *Energy for Computation*—energy spent on performing computations i.e., processing of data by devices in the network. This aspect is split into *General*, *Model*, and *Param*. just like in the *Energy for Communication* column.
(5) *Energy-saving Methods*—the optimization of fog using energy as an objective and/or constraint (the *Opt*imization column) and the utilization of technologies minimizing energy consumption (the *Tech*nologies column). The *Multi*ple column refers to works which describe multiple optimization problems and/or propose multiple methods for solving them. It is ticked, when the survey paper contains analysis of all optimization problems and methods from the surveyed works.

**Table 1:** Summary of survey works on fog computing—the energy perspective.

Research Work (Chronological Order)	Work Focus (Group)	1. Energy Consumption	2. Energy–Fog	3. Energy for Communication			4. Energy for Computation			5. Energy-Saving Methods		
		of Fog in General	vs. Cloud	General	Model	Param.	General	Model	Param.	Optim.	Tech.	Multi.
Yi et al. [15] (2015)	Current issues and future work directions (a)	∘	-	-	-	-	-	-	-	-	∘	-
Mao et al. [16] (2017)	Computation and communication models and resource management (c)	+	∘	+	+	-	+	+	-	+	+	-
Taleb et al. [17] (2017)	Network architecture, orchestration, and underlying technologies (a)	+	-	+	-	-	-	-	-	+	-	-
Hu et al. [18] (2017)	Key technologies of fog computing (a)	+	∘	+	-	-	+	-	-	+	-	-
Jalali et al. [10] (2017)	Power consumption, network design (a)	+	+	+	-	∘	+	-	∘	+	∘	-
Abbas et al. [19] (2018)	Extensively covers multiple areas (c)	+	-	+	-	-	+	-	-	+	-	-
Nath et al. [9] (2018)	Extensively covers multiple areas (c)	+	-	+	-	-	+	-	-	+	-	-
Mouradian et al. [20] (2018)	Categorizing and reviewing articles on architectures and fog-related algorithms (c, d)	+	∘	+	-	-	+	-	-	+	-	-
Mahmud et al. [21] (2018)	Taxonomy, resource and service provisioning (a, b)	+	-	-	-	-	-	-	-	-	-	-
Mukherjee et al. [22] (2018)	Fog computing architectures and models (b, c)	+	∘	+	+	-	+	+	-	+	-	-
Svorobej et al. [23] (2019)	Simulating fog and edge computing (d, e)	+	-	+	∘	-	+	-	-	+	-	-
Yousefpour et al. [24] (2019)	Comparison of fog computing and similar paradigms, taxonomy (b, c)	+	-	+	∘	-	+	-	-	+	∘	-
Moura and Hutchison [25] (2019)	Usage of Game Theory in wireless communications (d, e)	+	-	+	-	-	+	-	-	+	∘	-
Hameed Mohsin and Al Omary [26] (2020)	Energy saving and security in IoT and Fog (e)	+	-	+	-	-	+	-	-	+	∘	-
Qu et al. [27] (2020)	QoS and energy optimization in fog and other computing paradigms (d)	+	+	+	+	-	+	-	-	+	∘	-
Rahimi et al. [28] (2020)	Fog computing utilized by smart homes (e)	+	-	-	-	-	-	-	-	+	-	-
Caiza et al. [29] (2020)	Fog computing at industrial level (e)	+	-	-	-	-	-	-	-	+	∘	-
Bendechache et al. [30] (2020)	Simulations of resource management in cloud, fog, and edge (d, e)	+	-	-	-	-	+	-	-	+	-	-
Qureshi et al. [31] (2020)	Resource allocation schemes for real-time, high-performance computing (d, e)	+	-	-	-	-	+	-	-	+	+	-
Habibi et al. [32] (2020)	Various architectures of fog, e.g., reference, software, security (a, b)	+	+	-	-	-	-	-	-	+	-	-
Aslanpour et al. [33] (2020)	Comparison of performance evaluation metrics for cloud, fog, and edge (b, d)	+	+	-	-	-	+	-	-	+	-	-
Lin et al. [34] (2020)	Computation offloading—modeling and optimization (d)	+	∘	+	+	-	+	+	-	+	+	-
Asim et al. [35] (2020)	Computational intelligence algorithms in cloud and edge computing (d, e)	+	+	+	-	-	+	-	-	+	-	-
Fakhfakh et al. [36] (2021)	Formal verification of properties of fog and cloud networks (d, e)	+	-	-	-	-	-	-	-	-	-	-
QingQingChang et al. [37] (2021)	Health monitoring with fog-based IoT (e)	+	-	-	-	-	-	-	-	∘	-	-
Alomari et al. [38] (2021)	Taxonomy of SDN-based cloud and fog (b, d)	+	+	-	-	-	+	-	-	+	-	-
Ben Dhaou et al. [39] (2021)	Technologies and implementation of E-health using edge devices (e)	+	∘	+	-	-	+	-	-	+	+	-
Laroui et al. [40] (2021)	Extensively covers multiple areas (c)	+	+	+	-	-	-	-	-	+	-	-
Hajam and Sofi [41] (2021)	Smart city applications using IoT, fog, and cloud (e)	+	+	+	-	-	+	-	-	+	∘	-
Mohamed et al. [42] (2021)	Smart city applications using IoT, fog, and cloud (e)	+	+	+	-	-	+	-	-	+	∘	-
Martinez et al. [43] (2021)	Design, management, and evaluation of various fog systems (d)	+	-	-	-	-	-	-	-	+	-	-
Kaul et al. [44] (2022)	Nature-inspired algorithms for computing optimization (d, e)	+	∘	-	-	-	+	+	-	+	∘	-
Al-Araji et al. [45] (2022)	Fuzzy logic and algorithms in fog computing (d, e)	+	∘	-	-	-	-	-	-	+	-	-
Rahimikhanghah et al. [46] (2022)	Resource scheduling methods in fog and cloud (d)	+	∘	-	-	-	+	-	-	+	+	-
Tran-Dang et al. [47] (2022)	Resource allocation in fog using reinforcement learning (d, e)	+	+	+	-	-	+	∘	-	+	-	-
Acheampong et al. [48] (2022)	Computation offloading—metric, factors, and algorithms (d)	+	∘	+	-	-	+	-	-	+	∘	-
Goudarzi et al. [49] (2022)	Scheduling, taxonomy of application, and environmental architectures (b, d)	+	∘	-	-	-	-	-	-	+	∘	-
Ben Ammar et al. [50] (2022)	Computation offloading and energy harvesting (d, e)	+	∘	+	+	+	+	∘	-	+	+	-
Dhifaoui et al. [51] (2022) (e)	Edge, fog, and cloud computing in smart agriculture	+	+	+	-	-	+	-	-	+	-	-
Sadatdiynov et al. [52] (2023)	Optimization methods for computation offloading (d)	+	∘	+	+	-	+	∘	-	+	∘	-
Avan et al. [53] (2023)	Scheduling for delay-sensitive applications (d)	+	+	+	-	-	+	-	-	+	-	-
Zhou et al. [54] (2023)	Computation offloading, overview of MEC (a, d)	+	∘	+	-	-	+	-	-	+	-	-
Patsias et al. [55] (2023)	Optimization methods for computation offloading (d)	+	-	+	-	-	+	-	-	+	∘	-
Shareef et al. [56] (2024)	Handling imbalanced IoT data with fog (d, e)	+	-	+	-	-	-	-	-	+	-	-
Our work (2024)	Energy consumption in fog—modeling, parameterization, and optimization (d, e)	+	+	+	+	+	+	+	+	+	+	+

The survey works are chronologically ordered in Table 1 and are classified there in the following groups:

(a) *Overview and architecture*—this category encapsulates works that present the general view of fog computing and present some of its current applications and challenges. It includes the works [10,15,17,18,21,32,54]. Among them, the one most focused on energy consumption is [10].
(b) *Taxonomy and comparison with similar paradigms*—works in this category focus on presenting similarities and differences between fog computing and other similar paradigms, e.g., cloud computing, edge computing, and Mobile/Multi-Access Edge Computing (MEC). Works in this category include [21,22,24,32,33,38,49]. All of them also belong to other categories.
(c) *Broad and deep survey*—works in this category thoroughly examine multiple topics. These works survey numerous sources—over 100 in the case of [19,20,40] and over 200 in the case of [9,16,22]. The work [24], with over 400 cited works, perfectly fits into this category. It also fits into the category *Taxonomy and comparison with similar paradigms*.
(d) *Resource allocation and optimization*—this category refers to works that study the management of fog networks, resource allocation strategies, and various optimization algorithms. Works that broadly survey resource allocation and optimization include [20,27,34,43,46,48,52,54,55]. Other works are more focused on particular scenarios—Alomari et al. [38] study resource allocation in fog and cloud based on SDN, while Ben Ammar et al. [50] examine energy harvesting edge devices, Avan et al. focus on delay-sensitive applications in [53], and Qureshi et al. focus on high-performance computing in [31]. There are also works that focus on particular solutions to the optimization problems: nature-inspired algorithms in [35,44], algorithms based on fuzzy logic in [45], Reinforcement Learning (RL) algorithms in [47], and algorithms based on game theory in [25]. Bendechache et al. and Svorobej et al. focus on the simulation environments rather than the resource management problems themselves in [23,30], while Aslanpour et al. provide a set of performance metrics that can be used in network optimization [33]. Goudarzi et al. examine scheduling applications while providing a taxonomy for application and network architectures [49]. Fakhfakh et al. perform the formal verification of network properties and optimal solutions in [36]. Finally, Shareef et al. [56] examine various aspects of processing imbalanced data generated by IoT devices.
(e) *Focused on particular topic*—there are multiple surveys focused on particular aspects of fog, which are not included in the previous categories: energy and security in fog and IoT [26], fog-based smart homes [28], fog computing at an industrial scale [29], health monitoring with edge devices/IoT [37,39], smart cities [41,42], and smart agriculture [51]. The focus of our work is complementary to them. The rest of the works in the category *Focused on particular topic* belong also to other categories and have already been discussed.

**Figure 2:**
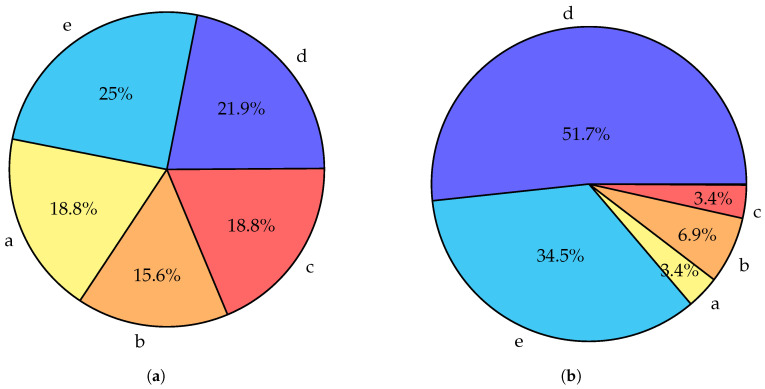
Charts summarizing topics of related survey articles examined in Section 5. a—*Overview and architecture*, b—*Taxonomy and comparison with similar paradigms*, c—*Broad and deep survey*, d—Resource allocation and optimization, e—*Focused on particular topic*. Works included in multiple categories are counted in multiple times. (**a**) Topics of surveys published in the years 2015–2020. (**b**) Topics of surveys published in the years 2021–2023.

It is interesting to point out how the topics of published surveys changed through the years. As shown in Figure 2, in the earlier years of fog research (up to 2020) all five major topic categories have similar representation. However, a comparison of results for works published post-2020 shows a significant change. Works focused on resource allocation and optimization (d) take up over half of all works in post-2020 compared to over one-fifth in pre-2020. The works specialized on particular topics (e) also increase their representation from one-quarter to one-third. Meanwhile, surveys classified by us as belonging to categories a—*Overview and architecture*, b—*Taxonomy and comparison with similar paradigms*, and c—*Broad and deep survey* see a significant decline in popularity. A few conclusions can be drawn from this visible trend. There is now less need for works discussing the taxonomy and architecture of computing paradigms as this work has already been performed. A decline in works in category “c” and simultaneous increase in “e” shows that works are becoming more focused over time. Finally, while the topic of optimization of fog networks has been popular for a long time, it is now as timely as ever.

### 2.2. Discussion of Overlapping Works

No existing survey paper fully covers the topics examined in this work, as shown in Table 1. Network modeling is rarely examined, while model parameterization is effectively never comprehensively surveyed. Only Jalali et al. briefly tackled this problem in [10], while leaving modeling details out in their work. It is especially true for modeling costs related to computations (covered in Section 3.5 and Section 4 of this work).

The works [16,34,50,52] have the highest overlap with our work (Table 1). Mao et al. examine various aspects of fog computing/MEC in [16]. They include the modeling of communication and computation, a comparison of works on resource allocation, multiple current and future issues, as well as standardization efforts. In contrast, our work views these models, resource allocation schemes, and issues through the lenses of energy consumption and saving. It is therefore narrower, albeit more focused. Our work examines works on resource allocation by extracting all necessary information related to studied optimization problems. We present it in a concise yet detailed manner including parameterization. Our approach is therefore complementary to [16], where only general descriptions of objectives and solutions are provided. Furthermore, no parameterization details of the models are provided in [16].

Ben Ammar et al. [50] provide communication models for task offloading and show the impact of wireless communication technologies on the parameterization of these models. They also examine multiple energy harvesting technologies in detail—down to the schematics of electrical circuits. However, in comparison with our work, their scope is narrow—they only examine works that combine both task offloading and energy harvesting, while we also consider works examining other applications of fog regardless of energy harvesting components. Moreover, unlike in our work, Ben Ammar et al. provide only two simple models describing computations in a task-offloading scenario. The authors of [50] do not provide the parameterization of these models.

Lin et al. [34] and Sadatdiynov et al. [52] consider fog/edge computing in the context of computation offloading only. Also, while they examine multiple works on optimization in the fog, Lin et al. and Sadatdiynov et al. only present a single problem and a single solution from each of the surveyed works. We, in contrast, analyze and present multiple optimization problems and proposed solutions from surveyed works. Eventually, the parameterization of models provided in studied primary works is not discussed in [34,52].

### 2.3. Scope of the Survey Discussion

Our work fully covers all topics shown in Table 1. It is particularly focused on providing a detailed analysis of energy consumption models used in surveyed works along with their parameterization. We compare and contrast approaches used by different authors to gain insight into which choices lead to which results.

Numerous works have shown in recent years that fog/edge computing (usually supported by a novel resource allocation scheme) can significantly improve network performance in terms of energy efficiency, delay, and other metrics. Our work offers the most comprehensive survey to date out of these works. Section 5.3 offers a concise view of various optimization problems through the lenses of objective functions, decision variables, and optimization methods. For works that present multiple optimization problems and/or multiple different solutions to the same problem, we examine and present all the problems and solutions, which differentiates our paper from existing surveys. Meanwhile, optimization methods are further examined in Section 5.4. We also consistently analyze the results of these methods and point out baseline solutions to which authors compare their methods.

On the other hand, we leave out several aspects of fog computing. We do not review privacy and security aspects, which are covered in the works [13,14,57,58,59,60]. We rather discuss energy costs of security and privacy measures in the fog as an important direction of future research. We also do not put emphasis on the disambiguation of the terminology used in the context of fog computing, which is the main aspect covered in [24,61]. For example, we survey works that study MEC networks without going into detail about the differences between various distributed computing paradigms. Our survey also does not include works that focus on electricity system networks and smart grids. We focus on the energy related to the transmission and processing of data rather than on utilizing the technologies behind fog, cloud, and IoT to optimize the electrical grid. Such approaches are studied in [62,63].

## 3. Modeling the Fog

We survey approaches related to (i) the modeling of the fog network, (ii) its application types, and (iii) tasks and incurred traffic in the first three subsections. The last two subsections are devoted to models of energy consumed for communication and for computation. Table 2 contains the main content of this section. We explicitly neglect works that do not provide models for energy consumption such as [64,65,66].

### 3.1. Network

**Figure 3:**
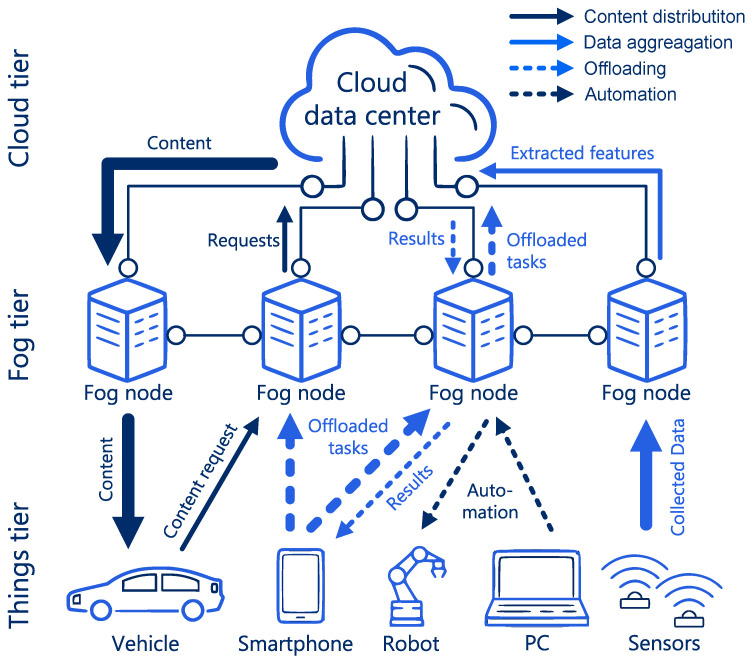
Different tasks performed by a fog network.

First, we compile the general view (in terms of terminology) of networks examined by works cited throughout our survey. The key aspect of fog is to provide resources (usually computational, but also networking and storage) close to the end users—close to the edge of the network. Throughout this work, we refer to elements providing these resources as Fog Nodes (FNs)—the term used in many works on fog including the reference architecture from [67]. Many surveyed works use other terms for nodes performing the same role, such as MEC server, edge node, or mobile cloud. Still, throughout this work, we describe them as FNs and call the tier (by some researchers called layer [9] or strata [20]) of the network, which consists of these FNs as a fog tier. It is the middle tier of the network out of three tiers, as shown in Figure 3 and explained in Section 3.3. The tier of the network that contains end users (such as sensors, MDs, smart vehicles, etc.) is called the things tier as in the IoT. Throughout this work, we consistently use the term MDs to refer to all these *things*. MDs can benefit from utilizing resources provided by the FNs. On the other side of the network, there is the cloud tier, consisting of resources from one or more cloud DCs. The cloud can also provide resources to users in the things tier.

Table 2 shows modeled networks through the lenses of costs (energy and delay) occurring in the network. The listed works (the first column of Table 2) are described with their authors and publication year. The *Application Type* column shows what kind of tasks are served by the network. The application types are explained in Section 3.2.

**Table 2:** Comparison of models—where (E)nergy is spent and (D)elay incurred. N/A stands for Not Applicable, “–” stands for no costs.

Work	Application	Communication			Computation			Discrete	Comments
	Type	Things-Fog	Intra-Fog	Fog-Cloud	Things	Fog	Cloud	Tasks/Continuous Workload	
1. Energy spent only by things—mobile devices									
Huang et al. [68] (2012)	Offloading	E and D	N/A	D	E and D	N/A	D	Tasks (e, f)	Single MD, no computing nodes in the fog tier.
Sardellitti et al. [69] (2015)	Offloading	E and D	N/A	N/A	N/A (*)	D	N/A	Tasks (a)	Inter-FN interference; local execution is mentioned multiple times yet no equations nor costs are provided.
Muñoz et al. [70] (2015)	Offloading	E and D	N/A	N/A	E and D	D	N/A	Tasks (c)	Single MD, separate modeling of Things-Fog UL and DL, single FN.
Mao et al. [71] (2016)	Offloading	E and D	N/A	N/A	E and D	–	N/A	Tasks (a)	Single FN, single MD, DVFS for MD.
Dinh et al. [72] (2017)	Offloading	E and D	N/A	D	E and D	D	D	Tasks (a)	Single MD; DVFS for MD; cloud is modeled not in conjunction with FNs, but in a separate scenario.
You et al. [73] (2017)	Offloading	E and D	N/A	N/A	E and D	D	N/A	Tasks (c)	Single FN.
Liu et al. [74] (2018)	Offloading	E and D	–	D	E and D	D	D	Tasks and Workload (c)	Social ties between MDs, single FN with multiple servers.
Feng et al. [75] (2018)	Offloading	E and D	N/A	N/A	E	–	N/A	Tasks (c)	Single FN.
Cui et al. [76] (2019)	Offloading	E and D	D	N/A	E and D	D	N/A	Tasks and Workload (a)	Single FN, multiple small cell BSs acting as relay nodes.
Kryszkiewicz et al. [77] (2019)	Offloading	E	N/A	N/A	E	–	N/A	Workload (a)	Single MD, single FN.
He et al. [78] (2020)	Offloading	E and D	N/A	N/A	E and D	D	N/A	Tasks (a)	
Shahidinejad and Ghobaei-Arani [79] (2020)	Offloading	E and D	D	D	E and D	D	D	Tasks (a)	Resource provisioning, edge gateways acting as relay nodes.
Nath and Wu [80] (2020)	Offloading and task caching	E and D	–	–	E and D	D	N/A	Tasks (a)	One or more FNs, FNs can cache tasks, FNs can fetch tasks from other FNs or cloud, fetching incurs costs but neither energy nor delay.
Bai and Qian [81] (2021)	Offloading	E and D	N/A	D	E and D	D	–	Tasks (a)	
Vu et al. [82] (2021)	Offloading	E and D	N/A	E & D	E and D	D	D	Tasks (a)	Includes direct transmission things-cloud.
Bian et al. [83,84] (2022)	Data aggr.—distributed training	E and D (*)	N/A	N/A	E and D (*)	N/A	N/A	–	Delay and energy spent are random in time, their models are not given; a single FN coordinating distributed learning; [84] is the longer version.
Yin et al. [85] (2024)	Offloading	E and D	N/A	N/A	E and D	D	N/A	Tasks (a)	single FN, single MD, DVFS for MD.
2. Energy spent only by fog nodes									
Ouesis et al. [86] (2015)	Offloading	–	E and D	N/A	N/A	D	N/A	Tasks (a)	
Xu et al. [87] (2017)	Offloading	D	–	E and D	N/A	E and D	D	Workload (c)	Transmission to cloud and processing in cloud are modeled jointly; single FN with multiple servers; resource provisioning.
Chen et al. [88] (2018)	Offloading	E and D	D	N/A	N/A	E and D	N/A	Tasks (a)	things–fog transmission costs spent by the FN.
Murtaza et al. [89] (2020)	Offloading	D	N/A	D	N/A	E and D	D	Tasks (a)	Service provisioning.
Gao et al. [90] (2020)	Offloading	N/A	E	–	N/A	E and D	–	Workload (c)	2 tiers of FNs, DVFS for FNs.
Vakilian et al. [91] (2020)	Offloading	N/A	D	D	N/A	E and D	D	Workload (c)	
Vakilian et al. [92] (2021)	Offloading	N/A	D	D	N/A	E and D	D	Workload (c)	
Vakilian et al. [93] (2021)	Offloading	N/A	D	– (*)	N/A	E and D	–	Workload (c)	Transmission to a cloud is mentioned throughout the model, but the mathematical description is missing.
Abdel-Basset et al. [94] (2021)	Offloading	N/A	–	N/A	N/A	E and D	N/A	Tasks (a)	Multiple VMs, each VM can be thought of as a separate FN.
Sun and Chen [95] (2023)	Offloading & Service caching	D	N/A	– (*)	– (*)	E and D	N/A	Tasks (a)	Includes non-energy costs paid by FNs. Local processing and transmission to cloud are mentioned without description.
3. Energy spent only by the cloud									
Do et al. [96] (2015)	Streaming	N/A	N/A	–	N/A	N/A	E	Workload (c)	Energy spent for computing by cloud refers to video processing, energy expressed in terms of carbon footprint.
4. Energy spent by nodes in multiple tiers of the network									
Deng et al. [97] (2016)	Offloading	–	–	D	N/A	E and D	E and D	Tasks and Workload (a, b)	Multiple clouds, DVFS for FNs and clouds.
Sarkar and Misra [98] (2016)	Offloading	E and D	–	E and D	N/A	E and D	E and D	Tasks and Workload (a)	
Zhang et al. [99] (2017)	Offloading	N/A	E and D	E and D	N/A	E and D	E and D	Workload (c)	Incomplete/ambiguous model descriptions due to magazine style.
Sarkar et al. [100] (2018)	Offloading & data aggr.	E and D	–	E and D	N/A	E and D	E and D	Tasks and Workload (a)	Additional energy cost due to infinite processing in cloud. Multiple VMs per FN, intra-FN, and inter-FN resource management.
Wang et al. [101] (2019)	Offloading	E and D	N/A	N/A	E and D	E and D	N/A	Tasks (a)	Includes transmission between MDs.
Sun et al. [102] (2019)	Content caching	E and D	N/A	E	N/A	N/A	E	–	Energy spent for computing by cloud refers to signal processing.
Kopras et al. [103] (2019)	Offloading	E and D	–	E and D	N/A	E and D	E and D	Tasks and Workload (a)	
Djemai et al. [104] (2019)	Offloading	E and D	E and D	E and D	E and D	E and D	E and D	Tasks (e, f)	
Abbasi et al. [105] (2020)	Offloading	–	–	D	N/A	E and D	E and D	Tasks and Workload (a, b)	Model taken directly from [97].
Roy et al. [106] (2020)	Offloading	E and D	–	–	N/A	E and D	E and D	Tasks (b)	2 tiers of FNs: dew and edge; each FN and cloud has multiple VMs; no modeling of intra-fog and fog–cloud transmission despite being shown as part of the system; includes failure & repair times of nodes
Wang and Chen [107] (2020)	Offloading	E and D	N/A	N/A	E and D	E and D	N/A	Tasks (a)	Single FN, DVFS for MDs and for an FN.
Kopras et al. [108] (2020)	Offloading	N/A	E and D	E and D	N/A	E and D	E and D	Tasks (d)	
Khumalo et al. [109] (2020)	Offloading	E and D	N/A	D	N/A	E and D	D	Task (a)	
Gazori et al. [110] (2020)	Offloading	D	D	D	N/A	E and D	E and D	Tasks (a)	2 tiers of FNs, multiple VMs per FN and cloud.
Zhang et al. [111] (2020)	Offloading	E and D	N/A	N/A	E and D	E and D	N/A	Workload (c)	Single FN, multiple RRHs.
Alharbi and Aldossary [112] (2021)	Offloading	E	E	E	N/A	E	E	Workload (a)	Two tiers of FNs.
Ghanavati et al. [113] (2022)	Offloading	E and D	–	N/A (*)	N/A	E and D	N/A (*)	Tasks (b)	Transmission between the MDs and broker (gateway or AP); broker–FNs transmission has no costs; transmission to a cloud is mentioned without description.
Kopras et al. [114] (2022)	Offloading	D	E and D	E and D	N/A	E and D	E and D	Tasks (a)	DVFS for FNs.
Kopras et al. [115] (2023)	Offloading	E and D	E and D	E and D	N/A	E and D	E and D	Tasks (a)	DVFS for FNs.
Jiang et al. [116] (2023)	Offloading	E and D	N/A	–	E and D	E and D	—	Tasks (a)	UAVs act like FNs, transmission through IRSs, additional flying-related energy costs for UAVs, DVFS for MDs and FNs, cloud coordinates the network.
Daghayeghi and Nickray [117] (2024)	Offloading	E and D	E and D	E and D	N/A	E and D	E and D	Tasks (a)	Scheduling nodes between MDs and FNs, multiple clouds.

**Figure 4:**
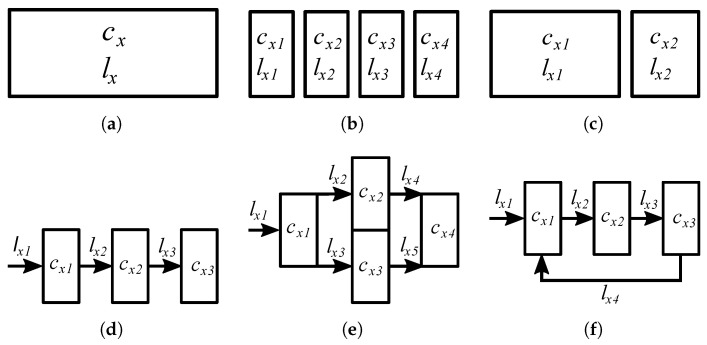
Comparison of different types of computational tasks. (**a**) Indivisible task. (**b**) Task divided into 4 subtasks. (**c**) Task for which any portion can be offloaded. (**d**) Sequential DAG. (**e**) General DAG. (**f**) Directed graph with a cycle.

The next three columns show whether the transmission of tasks occurs between nodes in given tiers and what costs related to this transmission are modeled. Depending on the work, “Intra-Fog” can include transmission between different FNs, between FNs and other nodes such as Remote Radio Heads (RRHs), and between servers/VMs within a given FN. Similarly, the next three columns show whether the processing (computation) of tasks occurs in these nodes. The term “Not Applicable (N/A)” means that in a modeled network, there is no possibility of (i) transmission between given nodes or (ii) computation by given nodes. “–”, on the other hand, means that there is transmission/computation, but the related costs are ignored or assumed to be negligible. “E” stands for energy consumption and “D” stands for delay caused by transmission or computation. We also add an asterisk “(*)” to the cells where the mathematical description of the model used by a given work does not correlate with the description in the text of said work. The *Discrete Tasks/Continuous Workload* column describes how the tasks performed by the network are modeled. The term “Discrete Tasks” means that there are individual tasks characterized by various parameters. The term “Continuous Workload” means that the tasks are modeled as part of the workload. The differences between these approaches are explained in Section 3.3. We also add letters in brackets (a, b, …) in Table 2 to show different types of modeled tasks in terms of divisibility as shown in Figure 4. The “default” examined network architecture includes multiple MDs, multiple FNs, and a single cloud. Deviations from this setup are included in the *Comments* column.

The existence of the things tier and the fog tier is either directly stated or implied in all surveyed works. N/A in the *Computation–Things* column does not mean that the things tier does not exist. It means that a given work does not consider the computation of requests by the things. On the other hand, multiple works do not consider the cloud tier at all (e.g., [111,113]). All but three of the works [68,83,96] consider one or more FNs with computing capabilities. Do et al. [96], Sun et al. [102] and Bian et al. [83] examine other applications than computation offloading. Meanwhile, Huang et al. [68] is the earliest of the works examined in Section 3 and it predates the named concept of fog computing [7]. While it is as an example of MCC, we leave it in Table 2 as it provides a valuable reference for comparison with works on fog computing.

We divide the works based on which devices spend energy in their models:

1. Only those in the things tier,
2. Only in fog,
3. Only in the cloud, as well as
4. Devices in multiple tiers.

All of the works in the group focused on the things tier consider energy spent on *Things–Fog Communication*. Only one of them ([77]) does not include the corresponding delay. On the other hand, only 5 out of 17 works in the same group consider communication delay between the fog and the cloud tiers. Four works out of those five additionally consider the delay caused by *Computation in the Cloud Tier*.

Works in the second group (*Energy Spent Only by Fog Nodes*) in Table 2 are not much more diverse in terms of coverage of energy and delay. Understandably, all but [86] cover energy and delay induced by *Computation in the Fog Tier*. *Computation in the Cloud Tier* is either not considered (four works), considered but only influencing delay (four works), or considered with its costs ignored (two works). Less focus is given to *Communication* with only one work each covering energy consumption and delay incurred by *Things–Fog Communication* ([88]), *Intra–Fog Communication* ([86]), and *Fog–Cloud Communication* ([87]).

Do et al. [96] is the only work that belongs to category 3. It considers energy spent on *Computation in the Cloud Tier*. Fog is modeled, but the energy consumed within the fog is neglected. Furthermore, delay is neglected in [96] for the whole network.

The final group encompasses works that model energy consumption within multiple network tiers. Consequently, it is the most diverse group. All but one work ([102]) consider energy consumption induced by *Computation in the Fog Tier*. A total of 5 out of 21 works consider costs (delay and energy) related to *Computation in the Things Tier*, and 16 out of 21 works consider those in the cloud tier (delay and energy in 13 cases, only energy in [102,112], and only delay in [109]). Eventually, we point out that only one work ([104]) covers energy consumption and delay incurred at all tiers and incurred by both communication and computation. Two other works ([115,117]) miss only energy and delay costs related to *Computation in the Things Tier*.

### 3.2. Application Type

Three broad types of services that a fog network can provide include offloading, content distribution, and data aggregation, as shown in Figure 3. These concepts are detailed in the second column of Table 2.

#### 3.2.1. Computation Offloading

Task offloading means transferring the task to another device (or another processing unit within the device) for processing. The idea to offload tasks from a device with limited capabilities to a more powerful one is not new. Experimental results from 1998 show how to increase the battery life of a laptop by putting some of its computational load to a nearby Base Station (BS) [118] or another laptop [119]. In the context of fog, offloading typically involves devices from the things tier sending computational requests to one or more nearby FNs. Upon receiving such a request, FN can process it itself or, depending on implementation, forward it to another FN or cloud. Finally, a processed task (result) can be transmitted back to its sender. As can be seen in Table 2, computation offloading is by far the most commonly studied use case for fog computing. Terms *resource provisioning* and *service provisioning* are sometimes used and describe the process of managing the network to assure the performance of offloading.

#### 3.2.2. Data Processing and Aggregation

In the case of offloading, the MDs offloading tasks can typically be seen as users who are interested in the results of processing offloaded tasks. This section is devoted to a group of applications called *data processing and aggregation*. The MDs—typically sensors—are the only source of data in this group. The data can be processed, aggregated, and stored in nodes in higher tiers of the network.

Works covering the topic of data processing and aggregation tend to focus on delay and do not take energy consumption into consideration. Such works are not included in Table 2. Examples include [120,121], which examine the transmission of health data to the cloud after being processed in an FN. Both works show that such an approach generates considerably less traffic and is faster than sending raw data to the cloud. Out of all works from Table 2, data aggregation is used only in [83,84,100].

#### 3.2.3. Content Distribution

Another broad group of applications features the users (MDs) accessing content from the network. For the last two decades, Content Delivery Networks (CDNs) have been used to quickly provide requested content using spatially distributed proxy servers [122]. Fog networks can act in a similar manner by caching content in FNs close to the end users. A particularly popular type of content distribution is streaming, i.e., the continuous transmission of multimedia files. Looking at the works from Table 2, task caching is performed in [9,80], service and content caching are performed in [95,102], while streaming is performed in [96].

### 3.3. Tasks and Traffic

Traffic modeling is crucial for modeling a network. While the topologies of the examined networks and types of applications served can differ significantly between the works in Table 2, there are many similarities in how the tasks performed by the network are modeled. Below, we describe the parameters describing the traffic and divide the works based on whether they model individual computational tasks or traffic as a continuous flow of data.

#### 3.3.1. Discrete Computational Tasks

The vast majority of works are concerned with the offloading of computations (Table 2). First, let us examine the tasks modeled discretely. Such computational tasks are usually parameterized by their size and their computational burden. Let $l_{x}$ be the size of task *x*, and let $c_{x}$ be the total number of computations required to process *x*. $l_{x}$ is given in bits or bytes. $c_{x}$ can be given in a number of Floating Point Operation (FLOPs) or other instructions, as well as a number of clock cycles. $c_{x}$ can be either given directly or calculated by multiplying $l_{x}$ by the arithmetic intensity $i_{x}$ of *x*:

(1) $$c_{x}=l_{x}i_{x}$$

$l_{x}$ is the main parameter influencing costs related to the transmission of *x* (discussed in Section 3.4), while $c_{x}$ influences the costs related to computations (Section 3.5). Finally, another parameter referring to the size of the task *x* is the size $r_{x}$ of the results transmitted back to the user. Many authors assume that $r_{x}$ is much lower than $l_{x}$ and ignore the costs related to the transmission of results altogether.

Another aspect of a computational task is its divisibility. We present various types of tasks in Figure 4. In many works (with one-task-one-node constraint as indicated later in the table in Section 5, tasks are indivisible. Each task has to be fully computed by a given node (Figure 4a). On the other hand, there are works in which each task can be divided into subtasks as in [106], and each of these subtasks can be transmitted to and computed at a different node (Figure 4b). Also, in some works, the tasks are infinitely divisible, as in [70,73,75,90], and in such cases, any portion of these tasks can be processed locally or offloaded (Figure 4c). This type of divisibility is often used in works that do not model individual tasks but rather continuous workload, like [91,92,93]. Finally, there are tasks that are divisible in a significantly more complex way. In [68,104,108], the tasks are multi-step computations with computational steps connected by inputs and outputs of varying sizes. These tasks are modeled as directed graphs, with each node representing a computational subtask and each vertex representing the size of the data. Each subtask can be computed by a different node but only in an order specified by the graph. Directed Acyclic Graphs (DAGs) are shown in Figure 4d (simple, sequential) and Figure 4e (more complex). Interestingly, while the authors of [104] describe their tasks as having a DAG structure, the example graphs used in [104] have cycles as in Figure 4f.

Delay tolerance $d_{\max,x}$ is sometimes used to describe the maximum delay tolerated for processing task *x*. This threshold can be hard, as in [73,78,80,97,107,114,115], which means that *x* has to be processed within this time. Alternatively, maximum delay can be a soft threshold, as in [101,104]. In these works, it means that exceeding the tolerated delay incurs a penalty—an additional cost.

$o_{x}$ denotes the origin of task *x*. In works that include MDs in their models, $o_{x}$ describes an MD that offloads (or processes locally) *x*. In works that do not model individual MDs, $o_{x}$ describes either the FN to which the *x* is originally offloaded or some intermediate node such as RRH.

#### 3.3.2. Continuous Computational Workload

Now, let us discuss the computational requests modeled not as discrete, individual tasks, but rather as a continuous workload. Computational workload refers to the amount of processing power or resources required to perform computational tasks. It is typically measured using various metrics, including Central Processing Unit (CPU) usage (in time, clock cycles or percentage), memory usage, input/output operations performed by a hard disk, the data volume to be processed or benchmarking metrics of the workload against some defined computing performance capacity. These metrics characterize the workload requested from a fog network. In general, they may take continuous values. Naturally, these values translate to the energy consumption of the network. We characterize some works in Table 2 as “Tasks and Workload”. In these works, there are parameters that characterize individual tasks (size, arithmetic intensity), but the delay and energy costs are calculated for the whole workload, ignoring individual tasks.

In [87], the arriving (requested) workload is considered as a whole (not arriving from discrete sources or end devices), although a discrete-time model is considered with a defined intensity and duration. The total arriving workload is counted in *units*. Likewise, in [91,92,93], the workload arriving at a fog node with a certain rate abstracts from the particular metrics describing it. It is treated as some continuous aggregated value. The decision on offloading is made by a central controller on the basis of the known processing power available at FNs and the arrival workload information.

In [98,100], the workload is considered as the data volume generated by the so-called Virtual Clusters (VCs), consisting of end devices and transported through the edge gateways toward the fog computing tier. Fog devices are also grouped to offer higher storage and computing capabilities. A group of fog devices is called an Fog Instance (FI). The workload is defined as a pair of values: the amount of data in bytes generated by a VC in a time slot and demanded to be served (processed) and stored. Additionally, service requests are classified as not requiring the cloud intervention (e.g., real-time services to be processed and served by FI) and requiring the intervention of the cloud computing layer (e.g., for analysis based on historical data sets and for long-term storage). Thus, the workload is given by the amount of aggregated data volume to be stored and processed with fog/cloud preference.

#### 3.3.3. Other Types of Tasks

It is worth noting that there are some examples of task request modeling that do not fit into the computational offloading models discussed in the previous sections. Bian et al. [83,84] examine a distributed learning scheme with a single FN and multiple MDs. There are no tasks to be modeled. The FN chooses MDs for training at each learning round. The chosen MDs spend some predetermined amount of energy on training if they are chosen. Do et al. [96] consider video streams from a cloud to FNs. This is represented by the amount of video streaming (like $l_{x}$) and a universal conversion factor from the size to computations (similar to $i_{x}$).

### 3.4. Model of Energy Spent on Communication

Many models presented in this section and in Section 3.5 show power consumption rather than energy. From these results, energy consumption can be calculated by multiplying power consumption by the time necessary to transmit/compute a given/average task. Some authors may avoid this step altogether and instead examine/optimize/show average power consumption.

#### 3.4.1. Wireless Transmission

The estimation of the power consumption in a wireless network is the crucial aspect of designing energy-efficient wireless communication systems. The power consumption model *P* presented in [123] consists of the power required for radio emission $P_{T}$ and the power consumed by the radio transceiver circuits $P_{C}$, which can be divided into power consumed by the baseband signal processing $P_{\mathrm{BB}}$ (both at the transmitter $P_{\mathrm{BB}-\mathrm{TX}}$ and at the receiver $P_{\mathrm{BB}-\mathrm{RX}}$) and by the intermediate-frequency and Radio Frequency (RF) signal processing $P_{\mathrm{RF}}$ (again both at the transmitter $P_{\mathrm{RF}-\mathrm{TX}}$ and at the receiver $P_{\mathrm{RF}-\mathrm{RX}}$):

(2) $$\begin{array}{c} P=P_{T}+\underset{P_{C}}{\underset{︸}{\underset{P_{\mathrm{BB}}}{\underset{︸}{P_{\mathrm{BB}-\mathrm{TX}}+P_{\mathrm{BB}-\mathrm{RX}}}}+\underset{P_{\mathrm{RF}}}{\underset{︸}{P_{\mathrm{RF}-\mathrm{TX}}+P_{\mathrm{RF}-\mathrm{RX}}}}}}. \end{array}$$

The power of transmission (radio emission) $P_{T}$ depends on the required coverage and channel conditions. The power consumed by the transmitter and receiver circuits $P_{C}$ depends on the transmission and reception techniques, applied technologies and standards, algorithms, implementations, etc. Three approaches to power consumption modeling are distinguished in the literature:

- High-level power consumption model,
- Measurement-based estimation,
- Transmitter and receiver components power estimation.

The high-level models can estimate the power consumption of many used technologies universally but at the cost of low estimation accuracy. Refs. [124,125] contain the most basic total power consumption model. The transmit power (dependent on the channel condition) and the constant circuit power (independent of the channel quality) comprise these studies’ overall power consumption models. A static term plus a dynamic term are included to model the power dissipation in a chip [126,127,128]. The latter is dependent on the supply voltage, clock frequency, and circuit capacitance, among other factors. The dynamic term, which is dependent on the clock frequency scales with the data rate. As a result, the attained data rate is linearly represented as the circuit power.

Measurements provide the basis of the second method for estimating wireless device power usage. High power estimation accuracy is guaranteed by this method; however, implementation, vendors, and equipment/link/network setup also play a major role. This method measures the overall power utilized, including the transmission power. As a result, the relationship between the transmit power and the circuit power cannot be ascertained a posteriori (after measurement), which implies that the transmission power allocation algorithm cannot be used with such models. Detailed power consumption data about a variety of commercially accessible devices are available in [129,130,131,132,133].

The most precise, but intricate, method is to assess the power usage of every transmitter and receiver component independently. Considering that the transceiver is integrated into a single chip, measuring any of its parts is barely possible. As a result, the architecture of each transceiver component is typically used to estimate its power consumption in the literature. The circuit-by-circuit power consumption model PC in this method is provided in [123]:

(3) $$\begin{array}{cc} P_{C} & =\underset{P_{\mathrm{BB}-\mathrm{TX}}}{\underset{︸}{P_{\mathrm{ENC}}+P_{\mathrm{MOD}}+P_{\mathrm{IFFT}}+P_{\mathrm{DAC}}}}\\ & +\underset{P_{\mathrm{BB}-\mathrm{RX}}}{\underset{︸}{P_{\mathrm{LPF}}+P_{\mathrm{ADC}}+P_{\mathrm{FFT}}+P_{\mathrm{DEMOD}}+P_{\mathrm{DEC}}}}\\ & +\underset{P_{\mathrm{RF}-\mathrm{TX}}}{\underset{︸}{P_{\mathrm{PA}}+P_{\mathrm{MIX}}+P_{\mathrm{LO}}}}+\underset{P_{\mathrm{RF}-\mathrm{RX}}}{\underset{︸}{P_{\mathrm{RFF}}+P_{\mathrm{LNA}}+P_{\mathrm{MIX}}+P_{\mathrm{LO}}}}, \end{array}$$

where $P_{\mathrm{PA}}$, $P_{\mathrm{LNA}}$, $P_{\mathrm{LO}}$, $P_{\mathrm{RFF}}$, and $P_{\mathrm{MIX}}$ are the power consumption of the Power Amplifier (PA), Low Noise Amplifier (LNA), Local Oscillators (LOs), RF filters, and a mixer, respectively. The baseband processing power includes the power consumption of the analog-to-digital converter $P_{\mathrm{ADC}}$, the digital-to-analog converter $P_{\mathrm{DAC}}$, the modulation $P_{\mathrm{MOD}}$ and demodulation $P_{\mathrm{DEMOD}}$, the encoding $P_{\mathrm{ENC}}$ and decoding $P_{\mathrm{DEC}}$, the low-pass filter $P_{\mathrm{LPF}}$, and when applicable, inverse fast Fourier transform $P_{\mathrm{IFFT}}$ and fast Fourier transform $P_{\mathrm{FFT}}$.

It is evident that a transceiver’s power consumption model might vary based on its construction. However, a few components are shared by the majority of digital transmission systems. These components that have the greatest share in the power consumption are presented and modeled in [134,135,136,137]. Reference [138] contains additional system-level energy models for the RF front-end components of a wireless transceiver with exemplary power consumption numbers from the majority of frequently cited works.

Further illustrative power consumption statistics are provided in the context of Long Term Evolution (LTE) technology in [139]. In [140,141], the power consumption models from the previously cited studies have been modified for the multi-user massive Multiple-Input and Multiple-Output (MIMO) situation. The model has been expanded to include components unique to the scenario being described.

The majority of the aforementioned articles concentrate on the power usage of the RF front-end and channel coding, ignoring the power used by other baseband signal processing techniques, which account for a sizable portion of power consumption when it comes to short links. This aspect is given additional attention in [142,143,144,145,146]. For example, the number of operations required to encode or decode the information bit for the channel coding techniques is available in [142,143]. The total power used by channel coding can then be calculated using the energy expenditure per operation.

Lastly, there is a trade-off between the power consumption models’ accuracy and their definitional difficulties. It is evident that a low representation of the actual system is found when the power consumption model is simple to construct. However, if the power consumption model’s accuracy is high, it can be very challenging to establish, for example, if all of the transmitter and receiver components are integrated into a single chip. As a result, the power consumption determined by the measurements and enhanced by stochastic modeling or interpolation appears to be a good solution.

#### 3.4.2. Wired Transmission

The power consumption spent on wired transmission is more predictable than the power consumption spent on wireless transmission due to the predictable (stable) communication channel. Wired transmission is realized in several parts of the fog network (Figure 3), i.e., rarely in the things tier, usually in the edge/fog tier, and essentially always in the cloud tier. Different transmission technologies are used throughout these tiers, e.g., GigabitEthernet in the things tier, Passive Optical Network (PON) in the edge/fog tier, and Wavelength Division Multiplexing (WDM) or Elastic Optical Network (EON) in the cloud tier. Power consumption $P_{wd}$ of a wired device depends on the load as follows

(4) $$P_{wd}=P_{idle}+\alpha \cdot P_{load},$$

where $P_{idle}$ is the power consumption of an idle device, α denotes load in the range <0;1>, and $P_{load}$ denotes power dependent on load [147]. $P_{idle}$ is relatively high with respect to the maximum power consumption of devices equal to $P_{idle}+P_{load}$. Power is consumed by devices located at network nodes and at network links:

1. Network nodes cannot be switched off or completely deactivated for connectivity reasons unless they are MDs and the end user decides so. However, parts of them related to links (wired transmission) can be adapted or even deactivated if the device has a modular structure. This refers to Adaptive Line Rate (ALR) in Ethernet networks [148] and data rate adaptation in EONs [149]. The modules need to be related to links (Network Interface Cards (NICs) in a router) in order to be dynamically switched between active and sleep states. Attempts are made to make $P_{idle}$ as small as possible as well as to change the profile of power dependency on load from a linear profile to a cubic one [150]. Dynamic Voltage and Frequency Scaling (DVFS) is the technology used behind these attempts.
2. Network links can be completely deactivated if there are alternative paths between the pair of nodes located at their ends. This provides higher power saving than link adaptation but comes with higher costs in terms of the calculation of alternative paths, traffic rerouting, and the time needed to activate and deactivate devices installed along the links (including NICs). Eventually, the reconfiguration process may lead to jitter and packets arriving out of order. Therefore, the reconfiguration has to be planned in advance with rather conservative traffic predictions and fair margins on link utilization [151].

### 3.5. Model of Energy Spent on Computation

Let us look at the energy consumption of device (node) *n* processing task *x*. The common way to model energy consumption is to take the number of computations $c_{x,n}$ performed by device *n* processing task *x* and multiply it by the unit cost $e_{n}$ of an instruction or a cycle.

(5) $$E_{n}\left(x\right)=c_{x,n}e_{n}$$

Similarly, the inverse $\frac{1}{e_{n}}$ (usually given in Floating Point Operations per Second (FLOPS) per Watt) can be used instead. What differentiates works is how the $e_{n}$ (or $\frac{1}{e_{n}}$) cost is parameterized. For some works, it is simply a value assigned to nodes in a network, e.g., to MDs in [75,79,82], to FNs in [88,98,100], and to cloud in [98,100,114]. A full list of works and used parameter values can be found in the table in Section 4.

Similarly, the energy cost $E_{n}\left(x\right)$ of node *n* processing task *x* can be achieved by multiplying the power consumption $P_{n}$ of node *n* by the time of execution of a given task $t_{x,n}$:

(6) $$E_{n}\left(x\right)=t_{x,n}P_{n}$$

as in [68] for MDs and [109] for FNs. The average time, including both execution and queuing, is used in [76,91,92,93]. Meanwhile, the authors of [113] write that their parameter denotes the energy consumption per cycle; however, they multiply it by the time of task execution.

For some works, there is no need to multiply power consumption by time to obtain energy as they only examine the levels of power consumption. Equation (7) shows an example of a model that substitutes the number of computations $c_{x,n}$ from Equation (5) with computational load (number of computations per second) $l_{x,n}$:

(7) $$P_{n}\left(x\right)=l_{x,n}e_{n}.$$

This is used for MDs in [77].

For all types of nodes, works often use a variation of Equation (8) to describe power consumption $P_{n}\left(u_{n}\right)$ of node *n* with relative processing usage/workload $u_{n}$ ($0\leq u_{n}\leq 1$):

(8) $$P_{n}\left(u_{n}\right)=\left(P_{n,\mathrm{peak}}-P_{n,\mathrm{idle}}\right)\cdot u_{n}+P_{n,\mathrm{idle}},$$

where $P_{n,\mathrm{peak}}$ and $P_{n,\mathrm{idle}}$ denote the maximum power and idle power of the node, respectively. It corresponds to Equation (4) used for wired transmission. Equation (8) or similar is used to describe the $P_{n}$ of cloud DC in [96], the $P_{n}$ of FNs in [89], and the $P_{n}$ of all nodes in [104,106]. Results from Equation (8) can then be multiplied by a certain time (e.g., time of execution of a given task) to obtain the energy cost.

The power models used in the surveyed works depend on the clock frequency and/or the number of servers as outlined below.

#### 3.5.1. Function of Clock Frequency—Nonlinear Models

Equations such as Equations (5) and (6) are general and rely heavily on parameters $e_{n}$ and $P_{n}$, respectively. They offer no insight into what influences their values. Below, we present a brief summary of models that consider the clock frequency as the main factor.

The power consumption of processors (Complementary Metal-Oxide Semiconductor (CMOS) powered) has three major components: *switching*, *short-circuit current*, and *leakage* [152], with *switching* being the main one. The switching power in a simplified form follows the formula

(9) $$P_{sw}=CV^{2}f$$

with *C* representing the total effective capacitance being switched per clock cycle, *V* representing voltage, and *f* representing clock frequency. Higher voltage allows the circuit to operate at a higher frequency by decreasing the delay [152]. Therefore, many works (all of the surveyed ones) look at power consumption as a function of frequency. DVFS is the technology that enables adjusting frequency and allows saving energy by operating at lower frequencies [153]. Assuming a linear relation between voltage and frequency, we end up with power being proportional to the cube of frequency ($P_{sw}\propto f^{3}$). However, higher operating frequency means that a given task can be computed faster, and the energy cost (the power multiplied by the computing time) is proportional to the square of frequency ($E_{sw}\propto \frac{P_{sw}}{f}\propto f^{2}$). One of these relations ($P_{sw}\propto f^{3}$ or equivalent $E_{sw}\propto f^{2}$) is used in most surveyed works using nonlinear energy models. They can be found in the middle column of row “Monomial” in Table 3. “Monomial” means that power/energy is modeled as a monomial function of frequency and therefore directly proportional to the frequency raised to a given power. On the other hand, works in the row “Polynomial” use polynomial functions of a given degree, i.e.,

(10) $$P_{sw}\left(f\right)=\sum_{k=0}^{n}a_{k}f^{k},$$

where $a_{k}$ are coefficients and *n* is the degree of the polynomial. As seen in Table 3, degrees other than three for power and two for energy are rarely used. Even then, in [114], while the model supports nth degree polynomial for modeling power of FNs, the scenarios used in the simulations assume the third degree. The cloud model used in [97] (and then cited in [105]) is not a typical polynomial, but in practice, it corresponds to a polynomial of the third degree with $a_{3},a_{0}>0$, $a_{2},a_{1}=0$.

In some works, e.g., [81,86,95,116], terms like processing power or computational capability are used instead of frequency.

Still, these parameters behave just like the clock frequency and are often symbolized by the letter *f*.

#### 3.5.2. Function of the Number of Servers

In some of the surveyed works [74,87,94,97,102,110], a computing node (FN or cloud) is modeled as a set of multiple servers (or processors/machines/VMs) that can operate independently. In some of these works, the power consumption depends on the number of active servers/processors of FN in [87] or in the cloud [97,102,105]. In [87], the authors only state that the power consumption of an FN increases with the number of active servers and with the size of the processed workload, but the actual formula describing it is not given. Later in the work, it is only specified that it is jointly convex in these variables.

### 3.6. Section Summary

There is a large variety of models used in works examining fog networks. The differences include the types of nodes present in modeled networks. MDs can process tasks in some works and cannot process them in others. Furthermore, some works do not include the cloud at all. There is a particularly large number of options chosen for FN-FN transmission: (1) no transmission as there is only a single FN; (2) no transmission despite multiple FNs; (3) instantaneous, no-energy-cost transmission; (4) instantaneous transmission with energy costs; (5) no-energy-cost transmission that incurs delay; and (6) transmission that incurs both delay and energy consumption.

Still, there are important similarities. A vast majority of surveyed works (43 out of 46) use computation offloading as a use case for fog. There is also general agreement that both communication and computation incur delay and energy costs. Most works model the computational requests as discrete, indivisible tasks.

Models for the power/energy consumption of particular equipment also vary. The widest range of models is used to model energy costs related to computing. Depending on the model, the values that can influence these costs include the number of computations, the computational load, the processor clock frequency, and the number of active servers.

## 4. Fog Scenarios and Parameterization

### 4.1. What Constitutes a Scenario?

**Figure 5:**
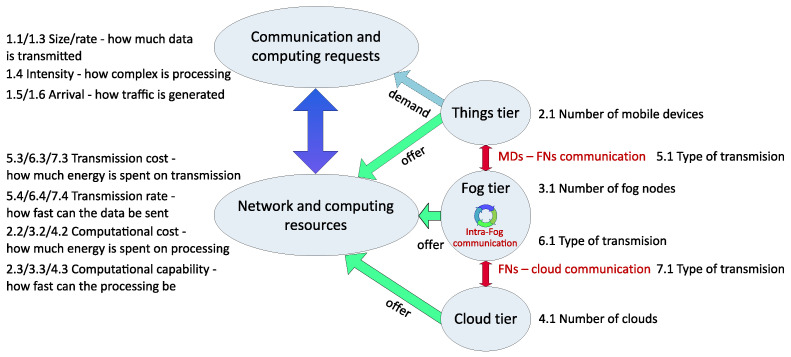
Common elements and parameters of the examined scenarios (parameter numbering inline with Table 4).

The surveyed works propose models that describe the mathematical foundations of how the fog network operates. There are many similarities between the chosen approaches, as shown in Section 3. What really differentiates works from one another are the values that authors choose to parameterize these models. The network performance (shown with metrics such as delay, energy costs, and others) always depends on the parameterization of nodes, connections between nodes, and traffic. Such a full set of parameter values defines a scenario under which a network is examined. Figure 5 shows certain key parameters defining a scenario and their relations. Meanwhile, Table 4 provides a comprehensive view of the parameterization used by the surveyed works.

Practically, every paper considered in this survey has a different set of testing parameters. However, the fog network scenarios are always parameterized with respect to two major fields: the communication and computing demand (generated tasks) and offer (network communications and computing resources). Our aim is to highlight the parameters that impact the network energy consumption or energy efficiency, which are the focus of this paper.

Communication and computing requests (tasks) stem from the things tier and are characterized by the size and rate (how many data are to be transmitted), intensity (how complex it is to process the request), and the arrival process (Figure 5). The three network tiers (characterized by the number of nodes in each tier) offer network and computing resources to serve requests. The networking resources depend on the type of transmission, transmission cost, and transmission rate, while computing resources depend on the computational cost and the computational capability. The parameters not shown in Figure 5 include the demand for particular content and the offering of that content, as well as demands and offers related to storing (rather than processing) data. We are also aware that one also cannot fully simulate real-world communication using only the transmission rate, especially if it is assumed to be constant. There are many physical phenomena (interference, noise, shadowing) and traffic related issues (jitter, packet loss) impacting it.

### 4.2. Scenario Parameterization—Comparison of Surveyed Works

Table 4 contains a concise comparison of scenarios examined in the surveyed works. It shows key parameters characterizing the networks and traffic. The majority of them are visualized in Figure 5. Table 4 is structured as follows. Its first part (1.) highlights the parameter values characterizing traffic in modeled networks. As discussed in Section 3.2 and Section 3.3, these are most often the tasks/workloads that can be offloaded to other nodes for processing. The next three parts refer to nodes, divided into three tiers (part 2 MDs, part 3 FNs, and part 4 cloud). The key parameters here represent the number of nodes and their computational capabilities and costs. The final three parts refer to communication between the nodes: part 5 between MDs and FNs, part 6 within the fog tier, and part 7 fog-to-cloud. We extract key parameter values that impact this transmission with a focus on energy costs.

#### 4.2.1. Classification Rules for Works in Table 4

Different works use different units. We convert them to the ones presented in Table 4 when applicable. This process often includes the multiplication/division of multiple parameters, e.g., division of FLOP/cycle by GFLOPS/W to obtain the nJ/cycle. Sometimes, we make assumptions as the surveyed work does not clearly state certain values. For instance, both data size and “computational load” are given in bits in [86], while arithmetic intensity or total required number of instructions/operations or cycles are never mentioned in [86]. This “computational load” (in bits) is divided by “computational rate” (in instructions per second) to obtain the delay. Therefore, we deduce that 1 bit corresponds to one instruction, which, in turn, corresponds to one clock cycle. The arithmetic intensity is therefore eight cycles/B. However, some works use units that cannot be converted in this way. This includes [87], where most parameters are defined in terms of abstract “units”, e.g., “units of power,” instead of Watts. For this reason, apart from [87], we also omit [91,92,93,97,105] from Table 4. We also omit [94,99] due to lack of explanation of many of the parameters used and the relations between them, as well as [109] presenting a model without assigning parameter values to it. We include [112], but not in the rows corresponding to transmission (5., 6., 7.). They separately model networks (LAN, MAN, WAN) as well as “edge/fog/cloud networking”. There is insufficient information in [112] to accurately report transmission parameters in Table 4. Finally, the works [83,96,102] do not fit into Table 4. Their modeled applications and examined scenarios differ significantly from the ones used in the rest of the surveyed works. Therefore, to avoid adding multiple works in the table that “fit” mostly into “N/A” and “unspecified” rows, we omit them entirely from the table. We also make a conscious choice not to add other sets of rows to Table 4, each referring to works [83,96,102]. All other works from Table 2 are present in Table 4.

Works present in the “None” row when referring to the number of nodes (i.e., MDs, FNs, and clouds reflected in rows 2.1, 3.1, and 4.1 in Table 4) are omitted from the rest of the corresponding table sections 2.2–2.3, 3.2–3.3, and 4.2–4.3. Similarly, works present in the “N/A” row when referring to the type of transmission (rows 5.1, 6.1, and 7.1) are also omitted from the rest of the corresponding table sections 5.2–5.6, 6.2–6.4, and 7.2–7.4. This is carried out to avoid clogging Table 4 with multiple additional “N/A” rows.

We use “Unspecified” in Table 4 when a parameter is missing in the surveyed work. In contrast, we use “Directly stated, no values given” when the work uses a particular parameter but does not state what value(s) is (are) assigned to it. Examples can be seen in [79], where the arithmetic intensity of task *i* is denoted by $\mu_{i}$, but no value is assigned to $\mu_{i}$. Similarly, the size of the task $L^{\mathrm{in},i}$ has a uniform distribution in the range <*a*, *b*>, but no values are assigned to *a* and *b*. Meanwhile “indirectly stated” means that a parameter value depends on values assigned to other parameters. A common example includes transmission rate, which is a function of transmission power (and other parameters like noise, interference, and channel bandwidth).

For parameter values describing the numbers of nodes used in surveyed works, we group them in corresponding rows. For other rows with numerical values, we write them just before the citation of the work. Multiple values are often written next to a given work. The notation {*a*, *b*, *c*} means that each of discrete values *a*, *b*, and *c* is used in the corresponding work. Meanwhile, <*a*, *b*> means that the values used belong to the closed interval from *a* to *b*; similarly, (*a*, *b*) and (*a*, *b*> mean open and semi-open intervals albeit their use is significantly less common. There are many different reasons why certain works use multiple different values for a given parameter. They include (i) different models of equipment used in a simulated scenario, e.g., two types of MDs in [104] having different computational capability {2.8, 4.5} Gcycle/s and cost {1.11, 1.14} nJ/cycle; (ii) different scenarios having different parameter setups—e.g., most plots in [103] show the results of fog network scenarios with an arithmetic intensity of 80 FLOPs/B, and some plots show results for scenarios with “easier” (8 FLOPs/B) and “harder” (800 FLOPs/B) tasks converted to 20, 2, and 200 cycles/B using 4 FLOPs/cycle value characterizing FNs in [103]; (iii) certain parameters being randomly generated at the beginning of the simulation, e.g., the computational capability of each FN is drawn from a uniform distribution <10, 15> Mcycles per second in [86]; (iv) certain parameters being randomly generated during the simulation, e.g., size and arithmetic intensity of computational tasks in [82]. The sizes of tasks are drawn from a uniform distribution <1, 10> MB, while the arithmetic intensities are drawn from one of ten uniform distributions starting from <100, 1000> cycles/B and ending with <1000, 1900> cycles/B with a step of 100 cycles/B. Results from simulations of scenarios with different intensity distributions are then compared with each other. Eventually, we round all the parameter values to three significant digits in Table 4.

#### 4.2.2. Differences and Similarities in Chosen Scenarios

Let us take a closer look at the content of Table 4. We start with part 1, which describes the demand parts of the scenarios. The vast majority of surveyed works examine tasks with either constant size or ones with sizes drawn from a uniform distribution (row 1.1). These sizes vary greatly between the works, from less than a kB to tens of MB. All four of the examined works in which tasks are divided into predetermined subtasks (row 1.2) choose a single-digit number of subtasks. Only three works that model the demand as a continuous flow of workload rather than discrete tasks fit into Table 4 (row 1.3). Arithmetic intensity (row 1.4), just like task size, is mostly chosen to be constant or random following a uniform distribution. Still, five works draw the sizes of tasks and the total number of computations per task from uniform distributions. This corresponds to arithmetic intensity being drawn from a quotient of two uniform distributions. Interestingly, the size and intensity of tasks are unspecified in five works, while the model is provided in 2 works, but failed to be specified with numerical values within the works. In [77], the arithmetic intensity is a simulation result rather than an input parameter (how high it should be for offloading to be energy efficient for given scenarios). Half (16) of the works assume that multiple tasks arrive in the network simultaneously (row 1.5). This allows them to develop algorithms optimizing the assignment of multiple tasks to nodes. Eight works assume that each task arrives one at a time, and eight works do not specify this. Meanwhile, when it comes to parameterizing the arrival distribution of tasks (row 1.6), a majority of the works do not provide the important information. Out of those that do, the majority choose the exponential distribution, while two works [79,90] use distributions based on real-world traffic.

In part 2 of Table 4, we examine the devices that constitute the lowest tier of a fog network—the MDs. While a few (6) works choose to focus on a single MD, the vast majority examine scenarios with multiple devices from several to over thousand, with [112] examining a scenario with 100,000 devices (row 2.1). Interestingly, three works do not model a single MD. They only look at the distribution of tasks within fog and cloud tiers. When it comes to computations performed by the MDs (row 2.2), a constant value describing the energy cost of processing is used in eight works and models where the value depends on the clock frequency of the device (see Section 3.5.1) are used in eight works. We add an asterisk (*) when describing the computational cost in [76] as the units used there are inconvertible. Ten works do not consider the possibility of MDs to process tasks, i.e.,, they are only the source of the demand and not of the offer. The computation capability of MDs (row 2.3) shows the same picture as their computational cost (row 2.2). The spread in values characterizing MDs (ignoring [74]) is between 0.1 and 7 nJ/cycle and between 0.1 and 5 Gcycles/s—less than two degrees of magnitude. It is significantly smaller than when it comes to task size/intensity, which have spreads of over four and five degrees of magnitude, respectively. Meanwhile, [74] is an exception—it parameterizes MDs as having only a 1.6–2.0 Mcycles/s capability—significantly less than other works. Their values for fog and cloud computational capabilities are also by far the lowest of the surveyed works. Moreover, the contrast in chosen computing cost 0.1 J/cycle = 1 × 10^8^ nJ/cycle is even greater. This 0.1 J/cycle value may be a result of a mistake as an MD running at 2 Mcycles/s with such cost leads to 200 kW power consumption. Five out of thirty-two works include mobility of the MDs as part of their models and scenarios (row 2.4).

The parameters of FNs (part 3 of Table 4) also significantly vary between the works. Unlike when it comes to MDs, more works (15) have a single FN, and only [68] considers no computational nodes in the fog tier (row 3.1). For the rest of the works, the numbers vary between several and multiple hundred. As for the processing of tasks (row 3.2), about half of the works (16 out of 35) do not consider energy costs carried out by the FNs. Of those that do, most (nine) assume that the costs vary depending on clock frequency, while three works use constant values. Random values (from a relatively narrow range) are used in [108] and no values are provided in [100,113] despite the parameters being directly stated. We add an asterisk (*) when describing the computational cost in [117] as the authors do not provide a clear relation between power consumption and capability of FNs, so the exact per-cycle costs cannot be computed. For computational capability (row 3.3), many works consider multiple different values for different scenarios and within scenarios. Few (3) assume that FNs have infinite capacity, which results in negligible (zero) computational delay.

Part 4 of Table 4 concerns the cloud. Half of the works do not consider a cloud at all (row 4.1). Reference [100] considers an eight-cloud DCs, [117]—3, and [112]—an unspecified number of DCs. All other works consider 1 DC. The computational cost per bit (row 4.2) is either ignored or assumed to be constant. Unlike in FNs and MDs, no works examine the cost as a function of frequency. The computational capability (row 4.3) is typically higher than in FNs and MDs, and assumed infinite in five works. For some works, there is a distinction between how fast a cloud can process a single task (per task capability) and the total capability. E.g., for works [103,114,115], the cloud can process any number of tasks simultaneously, but each at the rate of 1.5 Gcycles/s.

Separate sets of parameters are used when describing the things–fog–cloud communication in Table 4 parts 5 through 7. Communication between MDs and FNs is considered wireless (Wi-Fi/cellular, with varying levels of specificity) or not specified at all (row 5.1). Only a few (four) works do not consider any form of MD-FN transmission. Conversely, the majority of works (15) do not consider inter-fog transmission, and out of those that do, the majority model the connections as wired (row 6.1). As for fog–cloud transmission, about half of the works consider the connection as wired, and half of the works do not consider this transmission at all (row 7.1).

All but three works considering MD-FN transmission consider the energy costs related to it (row 5.3). The transmission power is either not included in the model (10 works), included as a function of other changing parameters (11), or directly stated (6), with values ranging from 10 mW to 2.51 W (row 5.2). For most (19) works, transmission cost per bit is indirectly stated—it depends on other parameter values such as channel gain and transmission rate (row 5.3). For two-thirds of works, the transmission rate also depends on other parameters. In the remaining one-third, the rate is directly stated and ranges from 0.25 Mbit/s to 1 Gbit/s (row 5.4). The vast majority of works do not include interference in their calculations (row 5.5) and assume that a given MD cannot choose FN to which it transmits data (row 5.6).

For the majority of works that include inter-FN communication, the energy costs are not considered at all (row 6.2). They are directly stated in [103,115,117] and indirectly stated in [86,90]. Transmission cost (row 6.3) is directly stated in more (five) works than indirectly stated, but it is usually assumed to have no cost (nine works). Transmission rates are typically higher than the MD-FN ones, ranging from 5 Mbit/s to 10 Gbit/s and six works assume an infinite rate (row 6.4).

Assumptions on the FN–cloud transmission rate (row 7.3) are made in eight works and range from 0.3 nJ/bit to 658 nJ/bit. No transmission cost is assumed in the majority of works (10). The stated transmission rates are even higher, reaching 40 Gbit/s (row 7.4). As far as energy is concerned, it is worth looking at the values that stand out. Reference [103] is the work where the power consumption of networking equipment is directly included (row 7.2). The two values used in [103] differ significantly because they describe different types of equipment: 5.5 W is the active power consumption of an Ethernet gateway and 6572 W describes a core network router (row 7.2). The work [117] stands out with the lowest power consumption and energy-per-bit cost. Meanwhile, scenarios examined in [82] allow for both FN–cloud and direct MD–cloud transmission (see rows 7.3 and 7.4). This direct transmission is wireless and characterized by significantly higher energy-per-bit cost than in any fog–cloud transmission. It is comparable to, albeit still higher than, the costs seen in wireless things–fog transmission in works [79,82,113]. In [80], like in most other works, the inter-FN and FN-cloud transmission incurs no delay nor energy costs. There are, however, other undefined costs related to these transmissions that are included in their models and scenarios.

### 4.3. Section Summary

The parameterization of a network model plays a crucial role in determining the network operation and calculated energy efficiency. Data compiled in this section shows that the chosen values vary greatly between surveyed works, spanning multiple orders of magnitude: size from 0.125 kB to 80 MB and arithmetic intensity from 0.125 cycle/B to 8000 cycle/B. This does not mean that some of these values are wrong, as different applications utilize the offloading of different kinds of tasks, e.g., a Portable Game Notation (PGN) file representing a chess game [155] is tiny (<1 kB) compared to a high-resolution image. However, we identify no standardized benchmarks commonly used by the researchers despite the fact that several benchmarks have been proposed in recent years [156,157].

Most works assign simple, discretionary values to characterize the traffic, e.g., constant or uniformly distributed sizes of tasks, and the Poisson process for the arrival of tasks. Only a few works [79,90] use real-world data for this characterization. Unsurprisingly, clouds are typically parameterized with higher computational capabilities than FNs, which in turn tend to have higher capabilities than FNs. This picture becomes less clear when it comes to the computational cost, particularly due to frequent ignoring of energy consumed by FNs and clouds for performing computations.

## 5. Optimization of Energy-Saving in the Fog

We first summarize fundamental terms related to optimization problem formulation (Section 5.1) and to optimization methods that can be used to reduce energy consumption in fog networks (Section 5.2). A detailed comparison of surveyed works through the lenses of optimization is provided in Table 5 and Section 5.3. We discuss the results achieved with optimization methods and applied technologies in Section 5.4.

### 5.1. Optimization Problem Formulation

The columns of Table 5 correspond to the design steps of an optimization problem [158], i.e., the definition of its objective function, decision variables, constraints, and scenario (parameterization). The first column states from which work a problem originates, and details of the notation used are discussed in Section 5.3. The last column is related to the methods used to solve the optimization problem.

In *Scenario* we broadly show what kind of application the network runs and what kind of problem (usually assignment of tasks to nodes) there is.

The *Objective function* is shown in the third column of Table 5. Examples of objectives include the minimization of energy consumption or delay or the maximization of some sort of utility function or user experience. In general, optimization problems can be single-objective or multi-objective. However, in all “multi-objective” surveyed works for [76], the authors choose to use a single-objective function that combines multiple objectives, usually a weighted sum. Throughout Section 5.1 and Section 5.2, we assume that the objective function is to be minimized, e.g., we discuss local/global minima and whether a function is convex. Maximization problems with objective function f(x) can easily be turned into minimization problems as follows: max(f(x))=−min(−f(x)).

We list the constraints that restrict the optimization in the third column. Formally, the *Constraints* are in the form of inequalities or equalities that certain parameters have to meet. While it is possible to formulate an unconstrained optimization problem, all surveyed works propose constrained problems in order to properly reflect the modeled network.

In the *Decision variables* column, we list the variables over which the optimization is performed. The goal is to find the values of decision variables that optimize the objective function. They can be discrete or continuous. The set of all possible decision variables is the search space of the problem.

The optimization problem is determined by the following characteristics [158]: smoothness, linearity, modality, convexity, and stochasticity. We do not discuss the characteristics of optimization problems proposed in surveyed works and do not label their classes, but we sometimes refer to the aforementioned characteristics throughout Section 5. Still, the vast majority of optimization problems from Table 5 belong to either either Mixed-Integer Non-Linear Programming (MINLP) or, more rarely, Mixed-Integer Linear Programming (MILP). Decision variables related to task allocation are usually discrete (including binary) and others (e.g., transmit power, operating frequency) are usually continuous.

**Table 5:** Approaches to optimization in the fog with respect to energy consumption.

SID. Work	Scenario	Objective Function	Constraints	Decision Variables	Optim. Methods
1. Convex optimization					
C1. Do et al. [96] (2015)	One cloud streaming video to multiple FNs	Maximize the utility (amount of video streaming) minus cost (cloud energy—carbon footprint)	Computational capacity of cloud	Amount of streaming to each FN	Proximal algorithm, ADMM
C2. Ouesis et al. [86] (2015)	Multiple FNs receiving tasks from MDs, FNs processing tasks or sending to other FNs	Minimize energy spent on the transmission of tasks by the FNs	Max. delay, FN computing rates	FN transmission power, allocation of computational resources to tasks	Reformulation into a convex problem, Lagrange method
C3. Sardellitti et al. [69] (2015)	Single MD offloading tasks to a single FN through a BS	Minimize energy consumption spent by the MD on transmission	Max. delay, max. MD transmission power	MD transmit covariance matrix, FN computing resources used	Reformulation into a convex problem, water-filling algorithm
C4. Sardellitti et al. [69] (2015)	Multiple MDs offloading tasks to a single FN through multiple BSs, multiple MDs transmitting without offloading	Weighted sum of MD transmission energy consumption	Max. delay for offloading MDs, min. rate for non-offloading MDs, max. MD transmission power	MD transmit covariance matrices, allocation of FN computing resources to tasks	SCA
C5. Sardellitti et al. [69] (2015)	Multiple MDs offloading tasks to a single FN through multiple BSs, multiple MDs transmitting without offloading (decentralized for each BS)	As in C4	As in C4	As in C4	SCA, separation of delay constraint in covariance matrices, dual decomposition
C6. Sardellitti et al. [69] (2015)	As in C5	As in C4	As in C4	Decomposition using slack variables, SCA with or without second-order information	
C7.1. Muñoz et al. [70]	One MD transmitting (UL) a task to a single FN	Minimize energy consumption spent on transmission by the MD	Min. transmission	MD transmission covariance matrix	Water-filling algorithm
C7.2	One MD receiving (DL) offloaded task from the FN	Maximize DL transmission rate	Max. FN transmission power	FN transmission covariance matrix	As in C7.1
C7.3	One MD offloading a task to a single FN, includes C7.1 and C7.2	Minimize total energy consumption spent by the MD	Max. delay, max. DL transmission power	Portions of tasks offloaded and processed locally, UL and DL transmission times	Problem reformulation in terms of portion of tasks offloaded and UL transmission rate, analytically finding opt. rate, finding opt. offloaded portion through gradient descent
C8. Muñoz et al. [70]	As in C7.3	Minimize total energy consumption spent by the MD	Max. DL transmission power	Portions of tasks offloaded and processed locally, UL and DL transmission times	Mathematically proving that partial offloading can never be opt. and finding the threshold above which local processing is opt.
C9. Dinh et al. [72] (2017)	One MD offloading tasks to multiple FNs or processing them locally	Minimize the weighted sum of latency and MD energy consumption	One-task-one-node	Allocation of tasks	LP relaxation
C10. Dinh et al. [72] (2017)	As in C9	As in C9	As in C9	As in C9	SDR
C11. Dinh et al. [72] (2017)	As in C10	As in C10	As in C10	Allocation of tasks, MD operating frequency	As in C10
C12. You et al. [73] (2017)	Multiple MDs offloading tasks (also partially) to a single FN with *infinite computing capacity* (TDMA) or processing them locally	Minimize the sum of energy consumption of all MDs weighted by unspecified fairness	Max. delay, one task per time slot	Size of offloaded portions of tasks, time slot allocation	Lagrange method, bisection search, this problem is used as a subproblem for other optimizations from [73]
C13. You et al. [73] (2017)	Multiple MDs offloading tasks (also partially) to a single FN (TDMA) or processing them locally	As in C12	As in C12	As in C12	Lagrange method, utilizing solution C12, 2D search for Lagrange multipliers (time slots, offloaded portions)
C14. You et al. [73] (2017)	Multiple MDs offloading tasks (also partially) to a single FN (OFDMA) or processing them locally	As in C13	Max. delay	Size of offloaded portions of tasks, subchannel allocation	Relaxation-and-rounding
C15. Feng et al. [75] (2018)	Multiple MDs offloading tasks (also partially) to a single FN or processing them locally	Minimize energy consumption of an MD with the highest consumption	Min. transmit rate, assignment of an IoT device to one and only one subcarrier	Subcarrier allocation, size of offloaded portions of tasks	Lagrange method, relaxation, subgradient projection
C16. Chen et al. [88] (2018)	Multiple MDs offloading load to FNs, FNs sharing load between each other—decentralized decision-making	Minimize delay over time	Max. energy cost over time, max. energy cost per time slot, max. delay per time slot	Task allocation to nodes (from one FN to another)	Lyapunov optimization
C17. Vakilian and Fanian [91] (2020)	Multiple FNs receiving offloaded workloads and processing them or sending them to other FNs or cloud for processing	Minimize weighted and normalized sum of energy consumption of FNs and delay	Processing rate of FNs	Allocation of workload to nodes	SCS
C18. He et al. [78] (2020)	A single MD offloading tasks to multiple FNs with potential non-colluding adversaries sensing its presence	Minimize energy consumption of an MD over time	Max. delay, max. task drop rate, max. likelihood of detection by adversaries, one-task-one-node	Task allocation to nodes, task dropping, MD transmission power	Lyapunov optimization
C19. He et al. [78] (2020)	A single MD offloading tasks to multiple FNs with potential colluding adversaries sensing its presence	As in C18	As in C18	As in C18	As in C18
C20.1 Gao et al. [90] (2020)	Multiple FNs receiving offloaded workload and processing it or sending it (or portions of it) to higher-tier FNs or cloud	Minimize average total power consumption of all FNs	Stability of queues, max. size of queues	Allocation of workload to nodes, FN transmit power, FN operating frequency	Lyapunov optimization, decomposition into C20.2, C20.3, and C20.4, workload prediction
C20.2	FNs processing workload	Minimize drift-plus-penalty for FN frequency decision	–	FN operating frequency	Polynomial (3rd degree) minimization
C20.3	Lower-tier FN wirelessly transmitting workload to higher-tier FN	Minimize drift-plus-penalty for transmit power decision	–	FN transmit power	Water-filling algorithm
C20.4	Lower-tier FN processing workload or sending it to higher-tier FN, higher-tier FN processing workload or sending it to cloud	Minimize drift-plus-penalty for offloading decision	–	offloading decision	Polynomial (1st degree) minimization
C21. Vu et al. [82] (2021)	Multiple MDs offloading tasks to multiple FNs or a cloud, or processing them locally	Minimize the sum of energy consumption of all MDs	One-task-one-node, max. delay	Allocation of tasks to nodes, uplink, downlink, and computing rates of cloud and of FNs	Relaxation-and-rounding
C22. Vu et al. [82] (2021)	As in C21	As in C21	As in C21	As in C21	Relaxation, improved BB algorithm
C23.1 Vu et al. [82] (2021)	As in C21	As in C21	As in C21	As in C21	FFBD into the following problems
C23.2	Multiple MDs offloading tasks to multiple FNs or a cloud, or processing them locally	Minimize the sum of energy consumption of all MDs	–	Allocation of tasks to nodes	Integer programming
C23.3	Checking if a solution to C23.2 is feasible	–	One-task-one-node, max. delay	Uplink, downlink, and computing rates rates of cloud and of FNs	None (verification of C23.2)
C24. Alharbi and Aldosdary [112] (2021)	Multiple MDs offloading tasks to two tiers of FNs (with lower tier nodes being called edge nodes) and cloud DCs	Minimize total power consumption	One-task-one-node, min. number of networking equipment such as routers, switches, terminals, and gateways	A total of 15 different variables	MILP
C25.1 Yin et al. [85] (2024)	Single EH MD offloading tasks to a single FN or processing them locally	Minimize the weighted sum of MD energy consumption and delay over time	One-task-one-node, battery level, stability of MD task queue	Task allocation to nodes, MD operating frequency, MD transmit power, harvested energy.	Lyapunov optimization, decomposition to subtasks C24.2 and C24.3
C25.2	Single MD optimizing EH	Minimize the product harvested energy and virtual level of the battery	Max. harvested energy	Harvested energy	Exhaustive search.
C25.3	Single MD offloading tasks to a single FN or processing them locally	As in C24.1	As in C24.1	Task allocation to nodes, MD operating frequency, MD transmit power.	Lyapunov optimization.
2. Heuristics					
H1. Huang et al. [68] (2012)	Single MDs offloading parts of tasks (directed graphs) to the cloud for offloading or processing them locally	Minimize energy consumption of the MD over time	Max. percentage of tasks exceeding delay constraint, stability of the system	subtask allocation to nodes	Lyapunov optimization, 1-opt local search algorithm
H2. You et al. [73] (2017)	Multiple MDs offloading tasks (also partially) to a single FN (TDMA) or processing them locally	Minimize the sum of energy consumption of all MDs weighted by unspecified fairness	Max. delay, one task per time slot	Size of offloaded portions of tasks, time slot allocation	Lagrange method, utilizing solution C12, greedy time slot allocation, 1D search for offloading Lagrange multiplier
H3. You et al. [73] (2017)	Multiple MDs offloading tasks (also partially) to a single FN (OFDMA) or processing them locally	Minimize the sum of energy consumption of all MDs weighted by unspecified fairness	Max. delay	Size of offloaded portions of tasks, subchannel allocation	Sequentially performing the following: assigning one subchannel to each MD according to priority, determining the total subchannel number and offloaded data size for each MD, assigning specific subchannels to MDs according to priority, finding the final offloaded data size for each MD
H4. Kopras et al. [108] (2020)	Single MD offloading tasks (directed graphs) to multiple FNs or cloud	Minimize the sum of energy consumption of all devices	Scheduling in nodes and links (one task per time slot), max. delay	Allocation of tasks to nodes	Clustering of nodes, exhaustive search over clusters
H5. Alharbi and Aldosdary [112]	As in C24	As in C24	As in C24	As in C24	Heuristic based on sequentially checking whether a task can be processed by an edge node, then FN, then cloud
3. Metaheuristics					
MH1. Djemai et al. [104] (2019)	Multiple MDs offloading tasks (directed graphs) to multiple FNs or cloud nodes, or processing them locally	Minimize the sum of energy consumption of all devices plus penalties for delay violations	Min. memory, min. computing capability	Allocation of tasks to nodes	Discrete PSO
MH2. Cui et al. [76] (2019)	Multiple MDs offloading tasks to an FN through relay nodes or processing them locally	Minimize energy consumption of MDs and delay (multi-objective)	One-task-one-node, processing rate of MDs and FN	Task allocation (binary—local or to the FN)	Modified NSGAII with task allocation as chromosomes and MDs as genes
MH3. Wang and Chen [107] (2020)	Multiple MDs offloading tasks to a single FN or processing them locally	Minimize total delay	One-task-one-node, max. task delay, max. task energy consumption	Task allocation to nodes, computation capability (operating frequency) of MDs and an FN	HGSA
MH4. Abbasi et al. [105] (2020)	Offloaded workload being shared between multiple FNs and clouds	Minimize fitness (a function of energy consumption and delay—not clearly stated)	Max. computing capacity of FNs	Allocation of workload to nodes, FN-cloud traffic rates, cloud on/off state, cloud operating frequency, number of turned on machines at cloud	NSGAII with assignment of workload to nodes as genes
MH5. Roy et al. [106] (2020)	Multiple MDs offloading tasks consisting of subtasks to two tiers of FNs or a cloud	Maximize fitness (combination of availability, delay, and power consumption)	Constraints included in the objective function	Allocation of subtasks to nodes	Adaptive PSO, GA
MH6. Vakilian et al. [92] (2021)	Multiple FNs receiving offloaded workloads and processing them or sending them to other FNs or cloud for processing	Minimize the weighted sum of normalized energy consumption of FNs and normalized delay	Processing rate of FNs	Allocation of workload to nodes	ABC
MH7. Vakilian et al. [93] (2021)	As in MH6	Minimize the weighted sum of energy consumption of FNs and delay adjusted by fairness (processing rate of a given FN divided by the total processing rate of all FNs)	As in MH6	As in MH6	Cuckoo algorithm
MH8. Abdel-Basset et al. [94] (2021)	Single FN allocating offloaded tasks to multiple VMs	Minimize the weighted sum of total energy consumption and the highest delay over VMs	One-task-one-node	Allocation of tasks to VMs	MPA
MH9. Abdel-Basset et al. [94] (2021)	As in MH8	As in MH8	As in MH8	As in MH8	Modified MPA
MH10. Abdel-Basset et al. [94] (2021)	As in MH8	As in MH8	As in MH8	As in MH8	Improved modified MPA
MH11. Ghanavati et al. [113] (2022)	Multiple MDs offloading sets of tasks to multiple FNs or a cloud	Minimize the weighted sum of energy spent by FNs and total delays for each set of tasks	One-task-one-node	Allocation of tasks to nodes	AMO
MH12. Daghayeghi et al. [117] (2024)	Multiple MDs offloading tasks to FNs or cloud DCs	Minimize total energy consumption and delay (multi-objective)	One-task-one-node, max. delay, computing capacity	Task allocation to nodes	SPEA-II modified with differential evolution
4. Machine Learning					
ML1. Xu et al. [87] (2017)	Single FN with multiple servers processing offloaded workload or sending it to a cloud for processing	Expected cost (delay, battery depreciation, backup power usage) over time with discount factor giving less weight to cost later in the future	Not clearly defined	Number of active servers in an FN, fraction of workload offloaded to the cloud	Utilizing RL to solve PDS-based Bellman equations
ML2. Wang et al. [101] (2019)	Multiple MDs offloading tasks to other MDs or an FN, or processing them locally	Minimize the weighted sum of energy consumption and delay, and exceeded delay penalties	MD battery level	Task allocation (wait, process locally, offload to an FN, offload to other MDs)	Deep RL scheduling
ML3. Wang et al. [101] (2019)	As in ML2	As in ML2	As in ML2	As in ML2	DDS, deep Q-learning
ML4. Nath and Wu [80] (2020)	Multiple MDs offloading tasks to one FN or processing them locally, FN caching previous tasks, FN fetching (downloading to cache) tasks from the cloud	Minimize the weighted sum of energy consumption, delay, and fetching cost over time	One-task-one-node, max. cached data size, max. delay	Allocation of tasks to nodes, caching decisions, fetching from cloud decisions, MD operating frequency, MD transmission power	Deep RL, DDPG
ML5. Nath and Wu [80] (2020)	Multiple MDs offloading tasks to one of multiple FNs (each MD can only offload to one FN) or processing them locally, FNs caching previous tasks, FNs fetching tasks from the cloud or other FNs	As in ML4	As in ML4	Allocation of tasks to nodes, caching decisions, fetching from cloud decisions, fetching from FNs decisions, MD operating frequency, MD transmission power	As in ML4
ML6. Bai and Qian [81] (2021)	Multiple MDs offloading tasks to multiple FNs or a cloud	Minimize the weighted sum of energy spent by MDs and delay	One-task-one-node	Allocation of tasks to nodes, allocation of channel resources to tasks, allocation of FN computing resources to tasks	A2C algorithm
5. Game Theory					
G1. Chen et al. [88] (2018)	Multiple MDs offloading loads to FNs, FNs sharing load between each other—decentralized decision-making	Minimize delay over time	Max. energy cost over time, max. energy cost per time slot, max. delay per time slot	Task allocation to nodes (from one FN to another)	Best-response algorithm
6. Mixed Approach					
MA1.1 Deng et al. [97] (2016)	Offloaded workload being shared between multiple FNs and clouds	Minimize the sum of energy consumption of FNs and clouds	Max. delay, max. computing capacity of FNs, max. FN-cloud traffic rate	Allocation of workload to nodes, FN-cloud traffic rates, cloud on/off state, cloud operating frequency, number of turned on machines at cloud	Approximation—decomposition into MA1.2, MA1.3, and MA1.4
MA1.2	Workload being shared between multiple FNs	Minimize the weighted sum of delay and energy consumption of FNs	Max. computing capacity of FNs	Allocation of workload to nodes	Convex optimization—interior-point method
MA1.3	Workload being shared between multiple clouds	Minimize the sum of energy consumption of clouds	Max. delay, max. computing capacity of clouds	Allocation of workload to nodes, FN-cloud traffic rates, cloud on/off state, cloud operating frequency, number of turned on machines at cloud	Non-convex optimization - generalized Benders decomposition
MA1.4	Workload being transmitted between multiple FNs and clouds	Minimize total transmission delay	–	FN-cloud traffic rates	Hungarian algorithm
MA2.1 Mao et al. [71] (2016)	Single EH MD offloading tasks to a single FN or processing it locally	Minimize weighted sum of delay and dropped task penalty over time	One-task-one-node, MD battery level	Allocation of tasks to nodes, MD transmission power, MD operating frequency, MD harvested energy	Proving opt. frequency should be constant for a task, using Lyapunov optimization to transform MA2.1 to a per-slot MA2.2 deterministic problem
MA2.2	As in MA2.1	Minimize weighted sum of virtual energy queue length, delay, and dropped task penalty over time	As in MA2.1	As in MA2.1	Finding opt. harvested energy with LP leading into MA2.3
MA2.3	As in MA2.2	As in MA2.2	As in MA2.2	Allocation of tasks to nodes, MD transmission power, MD operating frequency	Finding opt. values for frequency and trans. power, separately calculating costs for local execution, offloading, and dropping the task and choosing the lowest one
MA3. Liu et al. [74] (2018)	Energy harvesting MDs offloading workload (or portions of it) to a single FN (consisting of multiple servers) or cloud, or processing it locally	For each MDs minimize execution cost (delay + dropped task penalty) increased by weighted execution costs of other MDs in its “social group”	Stability of queues, battery level of MDs	Allocation of workload to nodes	Game theory—partial penalization, convex optimization—formulation of KKT conditions, convex optimization—semi-smooth Newton method with Armijo line search
MA4.1 Sun et al. [102] (2019)	Multiple MDs receiving content from a cloud through RRHs or D2D transmitters	Minimize the sum of energy consumption of all devices	–	On-off states of cloud processors, communication modes of MDs	Machine Learning—Deep RL
MA4.2	As in MA4.1	Minimize the sum of network-wide precoding vectors	Min. transmission rates, max. transmission power, computing resources of cloud	MDs data rates	Convex optimization
MA5.1 Zhang et al. [111] (2020)	Multiple MDs offloading load to one FN through RRHs or processing it locally	Minimize energy consumption divided by the size of processed tasks over time	Stability of queues	FN operating frequency (for computation and transmission), MD operating frequency, a fraction of offloaded load, FN transmission power, subchannel allocation	Lyapunov optimization, decomposition into MA5.2, MA5.3, MA5.4, MA5.5
MA5.2	Single MD offloading load to one FN through RRHs or processing it locally	Minimize Lyapunov drift-plus-penalty for the offloading decision	–	A fraction of offloaded load	Polynomial minimization
MA5.3	Multiple MDs offloading load to one FN through RRHs	Minimize Lyapunov drift-plus-penalty for the subchannel and transmit power allocation	–	FN transmission power, subchannel allocation	Game theory—two-side swap matching game, non-convex optimization—geometric programming
MA5.4	One FN processing offloaded load	Minimize Lyapunov drift-plus-penalty for FN computing resource allocation	–	FN operating frequency for computation and transmission	Convex optimization—decomposition
MA5.5	One MD processing tasks locally	Minimize Lyapunov drift-plus-penalty for MD computing resource allocation	–	MD operating frequency for computation	As in MA5.4
MA6. Kopras et al. [108] (2020)	Single edge node offloading tasks (directed graphs) to multiple FNs or cloud	Minimize the sum of energy consumption of all devices	Scheduling in nodes and links (one task per time slot), max. delay	Allocation of tasks to nodes	Heuristic—clustering of nodes, metaheuristic—discrete PSO
MA7.1 Bian et al. [83,84] (2022)	Single FN distributing parameters to and from multiple MDs in distributed training scheme	Minimize regret (a function of delay)	Wireless channel capacity, max. expected energy consumption of MDs, fairness—min. expected MD selection rates	Selection of MDs for training	Convex optimization—Lyapunov optimization, transformation into MA7.2
MA7.2	As in MA7.1	Minimize weighted sum of virtual queues (for energy consumption and fairness) and UCB term (estimated mean regret plus exploration cost)	Max. number of MDs the FN can communicate with in each round	Selection of MDs for training	UCB-based bandit algorithm, transformation into MA7.3
MA7.3	As in MA7.1	Minimize weighted sum of virtual queues	Selection of MD with the lowest UCB term, max. number of MDs the FN can communicate with during each round	Selection of MD for training	Heuristic—greedy approach
MA8.1 Kopras et al. [114] (2022)	Multiple FNs receiving tasks offloaded from multiple MDs and processing them or sending them to other FNs or cloud for processing	Minimize the sum of energy consumption of all devices	One-task-one-node, max. delay	Allocation of tasks to nodes, FN operating frequency	Decomposition into MA8.2 and MA8.3
MA8.2	An FN processing offloaded task	Minimize the energy consumption of the FN	Max. delay	FN operating frequency	Convex optimization—SCA, Lagrange method
MA8.3	Multiple FNs processing offloaded tasks or sending them to other FNs or cloud for processing	Minimize the sum of energy consumption of all devices	One-task-one-node	Allocation of tasks to nodes	Hungarian algorithm
MA9.1. Kopras et al. [114] (2022)	Multiple FNs processing offloaded tasks or sending them to other FNs or cloud for processing	Minimize the sum of energy consumption of all devices	One-task-one-node, max. delay	Allocation of tasks to nodes, FN operating frequency	Decomposition into 2 subproblems MA9.2 and MA9.3
MA9.2	An FN processing offloaded task	Minimize the energy consumption of the FN	Max. delay	FN operating frequency	Convex optimization—SCA, Lagrange method
MA9.3	Multiple FNs processing offloaded tasks or sending them to other FNs or cloud for processing	Minimize the sum of energy consumption of all devices	One-task-one-node	Allocation of tasks to nodes	Heuristic—greedy algorithm
MA10.1 Kopras et al. [115] (2023)	Multiple MDs offloading tasks to multiple FNs or a cloud	Minimize the sum of energy consumption of all devices	One-task-one-node, max. delay	Allocation of MD-FN transmission, allocation of tasks to nodes, FN operating frequency	Decomposition into 3 subproblems MA10.2, MA10.3, and MA10.4
MA10.2	An FN processing offloaded task	Minimize the energy consumption of the FN	Max. delay	FN operating frequency	Rational (3rd degree) function minimization
MA10.3	An MD offloading a task to one of multiple FNs	Minimize the sum of energy consumption of all devices	–	Allocation of MD-FN transmission for all possible computation allocations	Exhaustive search
MA10.4	Multiple FNs processing offloaded tasks or sending them to other FNs or cloud for processing	As in MA10.3	One-task-one-node	Allocation of tasks to nodes	Hungarian algorithm
MA11.1 Kopras et al. [115] (2023)	Multiple MDs offloading tasks to multiple FNs or a cloud	As in MA10.4	One-task-one-node, max. delay	Allocation of MD-FN transmission, allocation of tasks to nodes, FN operating frequency	Decomposition into 3 subproblems MA11.2, MA11.3, and MA11.4
MA11.2	An FN processing offloaded task	Minimize the energy consumption of the FN	Max. delay	FN operating frequency	Rational (3rd degree) function minimization
MA11.3	An MD offloading a task to one of multiple FNs	Minimize the sum of energy consumption of all devices	–	Allocation of MD-FN transmission	Heuristic—always transmitting with the lowest MD-FN cost
MA11.4	Multiple FNs processing offloaded tasks or sending them to other FNs or cloud for processing	Minimize the sum of energy consumption of all devices	One-task-one-node	Allocation of tasks to nodes	Hungarian algorithm
MA12.1 Sun and Chen [95] (2023)	Multiple MDs offloading tasks to FNs (each MD connected to a single FN) or processing them locally, FNs caching services	Maximize utility (price paid by MDs minus costs including energy) for the network provider	One-task-one-node, Max. FN power consumption, max. FN storage	MD transmission power, FN operating frequency, offloading cost (paid by and MD to an FN), cache location	Reducing incentive constraints, decomposition into MA12.2 and MA12.3
MA12.2	FNs processing offloaded tasks	Maximize utility	Max. FN power consumption	FN operating frequency	Convex optimization
MA12.3	Multiple MDs offloading tasks to FNs (each MD connected to a single FN) or processing them locally, FNs caching services	As in MA12.2	Max. FN storage	MD transmission power, cache location	Exhaustive generation of all possible cache locations, heuristic—greedy algorithm to find opt. cache locations, convex optimization—block-coordinate descent to find MD transmission power
MA13. Jiang et al. [116] (2023)	Multiple MDs offloading tasks to one or more UAVs acting like FNs	Minimize the weighted sum of energy spent by MDs and UAVs	One-task-one-node, max. computing capacity of MDs and UAVs	Locations of UAVs, task allocation to nodes, computing allocation to tasks, transmission matrix	Quantitative passive beamforming, machine learning –A2C algorithm with multi-head agent, multi-task learning; metaheuristic—LWS refinement

### 5.2. Optimization Method Classification

We show the optimization methods used to solve these problems in the column *Optim. methods* of Table 5.

#### 5.2.1. Optimization Method Attributes

The following attributes of optimization algorithms are listed in [158]:

*Order of information* shows how much information is given in the model. *Zeroth-order* means that there are only values of the objective function and constraints. *First-order* algorithms include information about gradients—first-order derivatives—of these functions with respect to the decision variables. Similarly, *second-* and *higher-order* algorithms include higher-order derivatives. None of the surveyed works specifically state that they provide gradients in their optimization problems. Still, multiple algorithms shown in Table 5 are gradient-based e.g., [75,114]. Typically, utilizing higher-order information makes an algorithm converge faster to the minimum. For example, in [69] the solution utilizing the second-order information is shown to be the fastest.

*Search*—the ways to search the design space can be broadly classified as either *local* or *global*. A global search spans the entire space to try to find the global minimum. In contrast, a local search starts at a certain point and from it, through a number of steps, tries to converge to a local minimum. If and only if a function is unimodal, then finding a local minimum is synonymous with finding a global one.

*Algorithm—Mathematical vs. Heuristic*—this division depends on whether the algorithm relies on provable mathematical principles or follows a practical method (heuristic) that is shown (but not proven) to be sufficient for a given problem. We find that this is the key difference between solutions to optimization problems proposed in the surveyed works. Therefore, in Section 5.2.2, we provide a summary of various families of mathematical and heuristic methods used in the optimization of fog networks.

*Function evaluation* can be *direct*—obtained by solving the provided models. In contrast, one can also generate *surrogate* models and optimize based on their evaluation. Surrogate models can be a result of interpolation or projection. The goal of using surrogate models is to build a model that fits the original one and is easier (faster) to optimize.

The *stochasticity* of the algorithm depends on whether it always (given the same initial conditions) evaluates functions at the same points and therefore converges to the same results. If that is the case, then the algorithm is *deterministic*. On the other hand, *stochastic* algorithms can evaluate different sets of points each time they are run according to some sort of random number generation. The surveyed works use many kinds of stochastic, and deterministic algorithms; e.g., all metaheuristics used are stochastic.

*Time dependence*—finally, the optimization problems can be divided into *static* and *dynamic* problems. Static problems involve solving the complete model at each optimization iteration. In contrast, dynamic problems involve solving a sequence of different problems as time progresses based on information that becomes available during this time. One can integrate the objective function over time to solve the problem for the entire time history and reduce a series of problems to a single one. Such an approach is commonly used as can be seen in Table 5—many authors use limits of expected values of objective functions and constraints as the time interval goes to infinity.

#### 5.2.2. Optimization Method Families

Table 5 shows that there is a multitude of different optimization methods used in the surveyed works. Similarly to [53], we group them into six broad families, and we discuss them in the next subsection.

*Convex optimization*: As mentioned in Section 5.1, convex problems can typically be easily solved by well-known methods such as interior-point methods or Newton’s method. Most convex optimization algorithms are mathematical (non-heuristic) and deterministic. The method of Lagrange multipliers and Karush–Kuhn–Tucker (KKT) conditions are used to directly include constraints in the objective functions. For networks modeled stochastically, Lyapunov optimization is used to stabilize the queuing times in the network while optimizing an objective such as minimizing energy consumption [78,90] or delay [83,88]. While Lyapunov optimization is the most commonly used one with convex optimization methods, it can also be used with other methods e.g., heuristics, in [68].

In some works, the proposed optimization problem is originally non-convex but then gets transformed (e.g., in [86]) or approximated into a convex one. A particular example of this approach is Successive Convex Approximation (SCA)—a technique that iteratively approximates non-convex functions by convex ones around given values as used in [114].

*Heuristics*: Heuristics are practical methods that are shown to provide good results (e.g., through trial and error) but are not mathematically proven to be optimal [158]. Many heuristics involve lowering the search space of possible solutions on the basis that the optimum is unlikely to be found in cut-off regions. They can also involve some “clever” approximations or adjustment of the order in which the problem is solved. An example whose variations come up in multiple works is the greedy algorithm, which relies on separating the problem into multiple stages and sequentially making the optimal choice at each stage (e.g., allocating a node sequentially for each task as in [114] or allocating time slots sequentially to each task in [73]).

*Metaheuristics*: “A metaheuristic is formally defined as an iterative generation process which guides a subordinate heuristic by combining intelligently different concepts for exploring and exploiting the search space, learning strategies are used to structure information in order to find efficiently near-optimal solutions” [159]. All metaheuristic algorithms shown in Table 5 are based on concepts of natural science, e.g., evolution—Genetic Algorithm (GA) as in [76] or animal behavior—Marine Predators Algorithm (MPA) as in [94]. They are typically model-agnostic and can be adapted to solve many different optimization problems. They belong to the heuristic category in a broader sense—their effectiveness is not proven mathematically. They are also predominantly stochastic, randomly adjusting parameters throughout the optimization.

*Machine learning*: Machine learning methods form another family of optimization techniques that can be distinguished. Surveyed articles mostly use variations of RL—agents learn to take actions maximizing the reward and deep learning—using neural networks with multiple layers. These works also belong to the broader heuristic category.

*Game theory*: In the majority of studied works, there is a common objective for the entire network. For optimization problems where each user/agent/node has its own goal, a solution based on game theory can be proposed as in [111,160]. These methods are not heuristic. They follow well-proven mathematical principles.

*Mixed approach*: Many optimization problems shown in Table 5 are split into subproblems. The solutions to these subproblems often utilize multiple algorithms from different families. We put them together in a separate part of Table 5 and examine these subproblems in separate rows.

*Non-convex optimization*: While it is generally easier to solve convex problems, there are also algorithms that solve various kinds of non-convex problems that do not fit into any aforementioned families. No optimization problem shown in Table 5 exclusively fits into this family. Therefore, the family is not included in Table 5. The authors of [97,111] use such methods for some of their subproblems.

### 5.3. Comparison of Works on Fog Optimization

We focus on energy/power in this survey. Therefore, we leave out works on optimization that do not have energy/power as part of their objective function or constraints like [110]. Also, we do not include works such as [77,79,89,98,100,103] that do not state any optimization problems, as defined in Section 5.1. These works show the results related to the operation of fog networks and perform parameter sweeps but do not perform the optimization. We also do not include [99] as it is written in a magazine style and does not include essential information (mathematical formulas) such as constraints and decision variables for energy optimization. Similarly, Khumalo et al. [109] present a simplified version of an optimization problem but do not provide their solution results, leaving them for future work. These works can still be found in Table 2. Optimization results are provided in the next work of Khumalo et al. [161]. However, the authors modify the model and optimization problem so that they no longer include energy consumption. Also, works [162,163,164,165,166,167,168,169,170,171,172,173,174,175] are only mentioned in this section as the origins of solutions chosen by the authors of other works for comparison. They neither model nor optimize energy consumption.

The summary of fog optimization problems and proposed solutions can be found in Table 5. It is structured in the following way. First, we name the work from which the optimization problem originates and describe the scenario under which the network operates. The solutions proposed in the works are marked with Solution IDentifiers (SIDs). In the next columns, we write the objective function, constraints, and decision variables for the optimization problem. Eventually, the proposed solutions are named.

We have multiple entries in Table 5 for some works. In some cases, a surveyed work proposes multiple different optimization problems. We then use multiple SIDs for problems within the same work. This includes the following works: [73]—three different optimization problems addressed by (i) C12, (ii) C13 and C14, and (iii) H2 and H3 respectively, [72]; C11 adds another decision variable to optimization problem compared with C9 and C10 [80]; and ML4 and ML5 also have different problems. In other cases, there are multiple solutions (often with varied levels of complexity) to the same problem. This includes the works [72,82,88,94,108,114,115]. Finally, some optimization problems are split into various subproblems, each solved with a different method. This includes the works [70,71,82,83,84,90,95,97,102,111,114,115]. These subproblems are assigned a SID with a sub-ID in Table 5. E.g., two distinct solutions (MA8 and MA9) are proposed in [114]. Each of them is split into subproblems (MA8.1, MA8.2, and MA8.3 for MA8, MA9.1, MA9.2, and MA9.3 for MA9) listed in Table 5.

In the *Constraints* column, we choose not to list “obvious” constraints on the decision variables listed next to it. For example, when a work has MD transmission power as a decision variable, we do not necessarily put maximum MD transmission power as a constraint nor the fact that it cannot be lower than zero. Similarly, we do not include constraints on allocation variables, e.g., which tasks are assigned to which node. The term “over time” is used in Table 5 and the rest of Section 5 to describe the expected value of a parameter over a time period as its length approaches infinity. Such an approach is often used in the surveyed optimizations (C16, C18, C19, H1, ML1, ML4, ML5, G1, MA2, MA5) for their objective functions. The authors do it to make their optimization problem deterministic and static. Decision variables “allocation of tasks/workload” take many forms in surveyed works. They can be binary (whether to offload) or continuous (what fraction to offload). They also decide which nodes tasks are offloaded to in works with multiple nodes that can process them. The abbreviations max., min., and opt. refer to maximum/maximal, minimum/minimal, and optimum/optimal, respectively.

Table 5 does not include baseline solutions used by the authors only for comparison with their proposed algorithms. Instead, we mention them while describing works in Section 5.4. Example baseline solutions include trivial algorithms such as random assignment or sending all tasks to the same node, or, on the other end of the spectrum, performing an exhaustive search.

### 5.4. Summary of Optimization Methods and Results

Below, we briefly summarize each work from Table 5 and discuss the results achieved by the authors. We also use this section to discuss some ambiguities present in the surveyed works. We divide the works by the role that energy consumption plays in their optimization problems. Within these subsections, they are ordered by year of publication, with the oldest publication being listed first. Please mind the different ordering (and classification) of works in this section and in Table 5.

#### 5.4.1. Energy as a Sole Objective

Huang et al. [68] examine a network with a single MD offloading parts of tasks (which are modeled as directed graphs) to the cloud (through a BS or Wi-Fi AP) or processing them locally. The optimization problem is to minimize the average energy consumption of the MD while satisfying the stability of the queues and keeping the percentage of tasks that fail to be executed within a given time below a certain threshold. The proposed solution (H1) uses the Lyapunov optimization and one-opt algorithm, which is based on a local search by changing only a single subtask allocation variable at a time. The authors compare their solution with the one from [162] and with two trivial baseline solutions: (i) sending all to the cloud and (ii) processing all tasks locally. Figure 4 from [68] compares the solutions and shows that the proposed one achieves the lowest energy consumption and the local processing has the highest energy costs. The proposed solution is compared with local processing in Figure 5 of [68] depending on the density of Wi-Fi network deployment. The results show that the denser the network, the more significant the drop in energy consumption is.

Ouesis et al. [86] examine a network with multiple FNs receiving tasks from MDs. They can process these tasks themselves or transmit them (or parts of them) to another MD for processing (the authors call this clustering). The original problem of minimizing energy consumption is non-convex due to the form of latency constraints. After reformulating the problem to be convex, it is solved through the Lagrange method. The proposed solution (C2) is compared with the following baseline solutions: no clustering—all tasks are processed in the FN they are sent to; static clustering—there is a predetermined way in which FNs share the computational load; and Successive Clustering which, is a greedy approach that optimizes each task sequentially. Counter-intuitively, the proposed solution has the highest energy costs. It stems from the fact that the baseline solutions (especially static clustering) have higher percentages of failure to meet the latency constraints of tasks. This effect intensifies with an increasing number of MDs offloading tasks.

Muñoz et al. [70] examine a network with a single MD offloading a task (also partially) to a single FN or processing it locally. Unlike other researchers, Muñoz et al. separately consider UpLink (UL) and DownLink (DL) transmission (C7.1 and C7.2, respectively) and show their optimization prior to proposing the main problem. The objective of the main problem is to minimize the energy spent by the MD while satisfying the task delay constraint. The solution (C7.3) involves redefining the problem with a smaller number of decision variables (two down from four) and convex optimization methods. They also propose a simpler scenario with no delay constraints in which a closed-form solution (C8) is found. The performance of the proposed solution is checked for different channel gains and delay constraint levels. Four transmission methods (e.g., MIMO 4 × 4) are checked. However, the only baseline solution to compare to is no offloading. The results show that better channel conditions, longer delay constraints, and higher MIMO levels decrease the energy consumption related to transmission and, in turn, the total energy spent by the MD.

Sardatelli et al. [69] examine a network with one or more MDs offloading tasks through one or more BSs to a single FN. Apart from these MDs, there can also be non-offloading MDs, which are included in the allocation of channel resources and the calculation of interference. The authors propose multiple different solutions to the non-convex problem of minimizing the energy consumption spent by the MD transmission. For a single-MD problem, an equivalent convex problem is proposed and solved with a water-filling algorithm (C3). Solutions to a problem with multiple MDs and FNs involve SCA. One centralized (C4) and multiple decentralized solutions are proposed. Decentralized solutions include one based on dual decomposition (C5) and one based on adding slack variables (C6). The latter one also has two versions, one of which utilizes the second-order information. The results achieved through the centralized solution are compared against a single baseline solution, where the FN computing resources are allocated proportionally. Decentralized solutions are compared against themselves. The centralized solution achieves lower energy costs than the baseline one. However, for tasks with high arithmetic intensity, the difference is negligible. All decentralized solutions converge to the same result, with the second-order one converging the fastest.

Deng et al. [97] examine a network with multiple FNs and clouds that share workload offloaded by the MDs. The problem is to minimize power consumption while keeping total delay below a certain level. The proposed solution (MA1) is achieved by approximating the problem (MA1.1) with three subproblems solving allocation in the fog tier (MA1.2), in the cloud tier (MA1.3), and choosing transmission parameters between the tiers (MA1.4) separately. Its effectiveness is not compared with any baseline solutions. The results show only the trade-off between delay and power consumption.

Gao et al. [90] examine a network with multiple FNs divided into two tiers and a single cloud. Lower-tier FNs receive an offloaded workload and can process it locally or send it (or part of it) to higher-tier FNs. Similarly, higher-tier FNs can process the workload themselves or offload it to the cloud. The proposed solution (C20) to an optimization problem predicts future workloads and is based on Lyapunov optimization. The authors examine the impact of multiple parameters on their solution: prediction window size, weighting the trade-off between power consumption and backlog in queues, and the number of higher-tier FNs chosen for possible allocation. It is compared against the following baseline solutions: no offloading, offloading of all workload to higher-tier FNs, offloading of all workload to the cloud, and random. All solutions converge to low power consumption (indistinguishable from 0 W and one another on the graph) with the all-to-cloud approach having the lowest power consumption at the beginning of the simulation. The proposed solution does not seem to optimize the objective function (average power consumption) any better than the simple baseline solutions. The fact that power consumption for all solutions appears to be converging to zero may be an artifact of the chosen model (see Table 2) in which e.g., transmission to the cloud and processing by the cloud incurs no delay and no power consumption.

You et al. [73] examine a network with multiple MDs and a single FN called edge cloud. MDs can offload their tasks or process them locally. They propose optimization problems for two types of access systems: Time Division Multiple Access (TDMA) and Orthogonal Frequency-Division Multiple Access (OFDMA). First, they solve a simpler problem (C12) with an FN of infinite capacity. For the TDMA system, they propose two algorithms: an optimal one requiring two-dimensional search (C13) and a heuristic suboptimal one (H2) allocating time slots to MDs using a greedy approach based on priority (function of channel gain and task arithmetic intensity). Equal allocation of time slots is chosen as the baseline solution. Results are shown for different time slot duration, FN computing capacity, and number of users. They show that suboptimal allocation results in energy costs close to those of optimal allocation. They both significantly outperform equal allocation under all examined scenarios. Their results show that energy costs grow much faster than linearly with the number of users. For OFDMA, they find an optimal solution (C14) based on relaxation and rounding. They also propose a faster suboptimal solution (H3) based on the sequential allocation of subchannels to MDs based on priority. As a baseline solution, they use greedy allocation, which does not consider the characteristics of offloaded tasks (size, arithmetic intensity). Results are shown for a different number of subchannels and number of users. One can see that suboptimal allocation causes energy costs close to that of optimal allocation, while costs achieved via greedy allocation are significantly higher. It is also clear that the time needed to find the optimal allocation is too high for applications in real-life scenarios, i.e., close to 6 min for 20 users on average.

Feng et al. [75] examine a network with multiple MDs and a single FN. MDs can offload their tasks or process them locally. Unlike most other works, they do not minimize total or average energy consumption but rather minimize the highest (worst-case) consumption of an MD. The baseline solutions include the full offloading of all tasks to the FN and the algorithms proposed in [68,70]. The solution proposed in [75] (C15, based on the Lagrange method and the subgradient projection) achieves lower energy consumption than [68] and significantly lower energy consumption than full offloading. Figure 3 from [75] shows that on average, C15 has higher energy consumption than the algorithm proposed in [70]; however, its worst-case energy cost is significantly lower. Interestingly, another plot (Figure 4 from [75] displaying results of a scenario with twice the number of subcarriers) shows that the algorithm from [70] achieves both lower average energy consumption and lower worst-case energy consumption.

Sun et al. [102] examine a network with multiple MDs receiving content from content servers through multiple different transmission paths including through RRHs or D2D transmission. The optimization problem is to minimize the overall energy consumption. The proposed solution (M4) is based on deep RL and convex optimization. Various parameter sweeps are performed to show their impact on total energy consumption. It is also contrasted with baseline solutions: random, Q-learning-based, D2D only, and cloud only. It achieves lower energy costs than all these baseline solutions.

Kopras et al. [108] examine a network with a single MD offloading tasks to multiple FNs and a cloud. The tasks are modeled as DAGs (see Figure 4) with each node in the graph representing a computational subtask that can be processed by another FN or cloud. The optimization problem is to minimize energy consumption while satisfying the delay constraints of tasks. Two solutions are proposed depending on the size of the network. Both utilize the clustering of nodes to decrease the search space. Next, one solution (H4) uses exhaustive search to find the optimal allocations for smaller networks, and the other solution (MA6) utilizes Particle Swarm Optimization (PSO) for larger networks. The chosen baseline solutions are exhaustive search and PSO based on [163]. In a smaller network, the proposed solution has close-to-optimal energy cost when compared with exhaustive search while being significantly faster to execute. In a larger network, the proposed solution (utilizing PSO) has lower energy costs, a shorter execution time, and lower percentage of tasks violating the delay constraint than “pure” PSO.

Vu et al. [82] examine a network with multiple MDs and multiple FNs. MDs can offload their tasks to the FNs or a cloud or process them locally. Unlike in other works, there is a possibility of a direct MD–cloud transmission. They propose three main solutions: one based on relaxation and rounding (C21), one based on Branch and Bound (BB) (C22), and one based on Feasibility Finding Benders Decomposition (FFBD) (C23). They are further subdivided: BB based on optimal node selection order (Local Fog Cloud or Local Cloud Fog) and FFBD into slow and fast variants. FFBD-slow/fast can also include resource allocation results from a relaxation-and-rounding solution. They also consider the following baseline solutions: all offloading and no offloading. The authors show results for a vast range of changing parameters. The no-offloading and the relaxation and rounding fail to find feasible solutions (satisfying latency constraints) in up to 90% of cases and up to 30% of cases, respectively. FFBD, BB, and all offloading always find feasible solutions, but all offloading tends to have significantly higher energy consumption. There is no straightforward answer as to which of the proposed solutions is the best at achieving the lowest energy consumption while FFBD-fast (with or without relaxation-and-rounding) tends to be the fastest.

Alharbi and Aldossary [112] examine a network with a large number of MDs (IoT sensors used in agriculture) offloading tasks to two tiers of FNs and cloud DCs. Lower-tier FNs are called edge nodes. Apart from computing nodes, the proposed model includes the power consumption of various networking equipment, including routers, switches, terminals, gateways, transponders, and amplifiers. The proposed solution (C24) uses an MILP solver. In the chosen scenario, the authors assume that 60% of tasks have low data rate and arithmetic intensity, 30% have moderate rate and intensity, and 10% have high data rate and intensity. The authors compare the power consumption of the network where tasks are processed at edge/fog/cloud nodes depending on their intensity to the one where all tasks are processed in the cloud. The results show that utilizing edge and fog nodes decreases power consumption and that Zigbee achieves the lowest consumption among simulated wireless network technologies. A simple heuristic algorithm (H5) is also proposed and is shown to achieve better results than the MILP solver. The authors do not explain how the results achieved with the heuristic can be better than the “optimal” results provided by the solver.

Kopras et al. [114,115] examine a network with multiple FNs and a cloud. In [114], the problem is to allocate tasks offloaded from MDs between FNs and the cloud and choose the operating frequencies for the FNs. Another decision variable is added in [115], i.e., choice of FNs for MD–FN transmission. The proposed solutions in both works (MA8, MA10) rely on sequential solving of subproblems and finishing with a matching problem solved by a Hungarian algorithm. Suboptimal, lower-complexity versions of their solutions (MA9, MA11) are also proposed with one part of the problem solved by a simple heuristic. The following baseline solutions are used in [114]: offloading all-to-cloud, offloading only between FNs, and no offloading from the FN that receives the task. The results show that, depending on the chosen parameters and compared with the baselines, the proposed solutions achieve lower energy cost, lower percentage of tasks unable to meet their delay constraint, or both. They show that the difference between results achieved by the full solution and the lower-complexity one is small and also highlight the gain from using DVFS. In [115], the authors also compare their solutions with no offloading and all-to-cloud and find that they achieve lower energy costs. The lower-complexity solution achieves virtually identical results to the full one for almost all input parameters. Both works verify that their solution finds the optimum when compared with an exhaustive search, and both works show that tasks with high arithmetic intensity are best served by the cloud and those with low intensity—by the FNs.

Jiang et al. [116] examine a network with multiple MDs, i.e., one or more Unmanned Aerial Vehicles (UAVs) which act as FNs, and a cloud. MDs can offload their tasks to UAVs or process them locally. Transmission utilizes Intelligent Reflective Surfaces (IRSs). The cloud gathers information about the network and manages resource allocation and positions of the UAVs. The objective is to minimize energy consumption spent by MDs and UAVs on transmission and computation, as well as on UAV hovering and flying. Their proposed RL-based solution uses the Advantage actor–critic (A2C) structure. It also uses a multi-head agent, multi-task learning, and action refinement based on Lone Wolf Search (LWS). The authors of [116] choose to examine the effects of each of these three techniques in their study. They show that a three-head agent achieves higher rewards than two-head and one-head agents. They also show that their multi-task learning method achieves constant energy consumption with an increased number of UAVs in the network, while this energy increases in the baseline solution from [164]. The authors claim that this constant cost is possible thanks to having memory in the system. They do not comment on how they are able to achieve this despite adding UAVs, which presumably spend energy on hovering and flying. The effectiveness of LWS refinement is measured against a taboo search [165] and no refinement. The proposed LWS refinement achieves slightly better results (lower loss function) than the taboo search and significantly better results than no refinement. Finally, the full solution is compared against three other deep RL algorithms from [166,167,176]. It achieves the lowest energy consumption, while the one proposed in [176] (called soft actor–critic) has the second lowest. It is also the fastest to execute.

Yin et al. [85] examine a network with a single EH MD and a single FN. MD can process its tasks locally (including keeping them in a queue or processing them at low frequency in standby mode) or offload them to the FN. The optimization problem is to minimize the expected value of a weighted sum of MD energy consumption and delay while maintaining the stability of the queue. The proposed solution is based on Lyapunov optimization and splits the problem into two subproblems: one subproblem focused on EH (C25.2) and one subproblem focused on task allocation (C25.3). They compare the results of their algorithm with two simple baseline solutions: greedy offloading and greedy local processing. They also use algorithms developed in [71] and in [177] for comparison. The proposed solution achieves the lowest cost (weighted sum of energy consumption and delay) by a factor of more than 10. As the amount of harvestable energy grows, the gap in the effectiveness of the proposed solution and that from [71] narrows.

#### 5.4.2. Energy as One of a Few Objectives

Do et al. [96] examine a network with multiple FNs and a single cloud. The cloud can stream video content to the FN providing utility to the end users in the process. The stated problem is to maximize this utility, subtracting costs related to power consumption in the cloud. This is solved iteratively using a proximal algorithm and Alternating Direction Method of Multipliers (ADMM) (C1). The authors do not compare their solution to any baseline solutions. All their plots show performance as a function of algorithm iterations.

Dinh et al. [72] examine a network with a single MD. It can offload its tasks to multiple FNs or process them locally. The results are shown for various numbers of tasks and nodes, as well as for varying weights assigned to delay and energy consumption in the objective function. Baseline solutions include exhaustive search, local processing, random assignment, and offloading all tasks to the cloud (which is added only in the model for this baseline solution). The Linear Programming (LP)-based algorithm (C9) causes higher costs (weighted sum of energy and delay) at larger numbers of offloaded tasks than the SemiDefinite Relaxation (SDR)-based algorithms (C10, C11), which achieve close to optimal solutions. They all significantly outperform local processing and random assignment. Comparison with the all-to-cloud case depends on the number of offloaded tasks—the lower the number, the bigger the difference in cost in favor of LP- and SDR- based algorithms.

Xu et al. [87] examine a network with a single FN and a single cloud. The FN is powered by battery, renewable energy, and non-renewable energy and can switch its servers between active and inactive states. It gets an offloaded workload and can process it in one of its servers or send it to the cloud. The problem of minimizing the total expected cost is formulated as a Markov Decision Process (MDP) and solved using RL. The proposed solution (ML1) utilizes Post-Decision State (PDS) and is compared with Q-learning, as well as with three simple schemes: one that utilizes all available battery energy in each time slot and two with fixed power consumption. The results show that the PDS-based RL achieves the lowest total cost, with Q-learning having the second lowest cost. Interestingly, both PDS-based RL and Q-learning have higher costs related to delay than the three other methods. However, they are significantly better at saving energy stored in a battery and minimizing costs related to usage of non-renewable energy, which offsets longer delays.

Liu et al. [74] examine a network with a single FN and a single cloud. There are multiple EH-capable MDs offloading tasks to an FN or a cloud (through FN) or processing them locally. MDs are connected by social ties, which influence the costs related to offloading. Their proposed solution (MA3) uses partial penalization to transform the original generalized Nash Equilibrium Problem (NEP) into a “classical” NEP. Then, using KKT conditions, they transform the problem into a system of non-smooth equations. Finally, they use the semi-smooth Newton method with an Armijo line search to solve the system. The authors focus on showing the impact of varying task-arrival rates on values such as task execution rates and costs spent on different parts of the network. The baseline solutions (whose results are shown only on a single plot) are taken from other works, namely [69,71]. Implementation details are not provided. Liu et al. show that their solution provides results with a lower average cost (penalties for delays and dropped tasks increased (penalized) by corresponding costs of socially linked MDs) than the solutions proposed in [69,71].

Cui et al. [76] examine a network with a single FN located at a BS and multiple small cell BSs, which relay tasks from MDs. MDs can also process tasks locally. The multi-objective problem (delay and MDs energy consumption) is solved using modified Non-dominated Sorting Genetic Algorithm II (NSGAII) (MH2). Results are plotted for various values of parameters: number of small-cell BSs, task arrival rate, MD transmission power, FN computing capability, and MD computing capability. They show the impacts on energy consumption, delay, and the trade-off between them. The authors do not compare the achieved results with any baseline solutions.

Wang et al. [101] examine a network with a single FN and multiple MDs. MDs can offload tasks to an FN or to another MD, or process them locally. The authors propose two solutions, one based on deep RL scheduling (ML2) and one based on Deep Dynamic Scheduling (DDS) with deep Q-learning (ML3). They choose the following baseline solutions: no offloading, offloading all tasks to other MDs, and offloading all tasks to the FN. The objective function is to minimize a weighted sum of energy consumption, delay, and penalties for exceeding task deadlines. Meanwhile, the results show energy and delay separately. The proposed solutions have lower energy costs than the baseline solutions with the “no offloading” one having the highest costs. On the other hand, “no offloading” and “offloading to MDs” achieve lower delay than the proposed solutions except for tasks with a large size, either in bits or number of instructions. From the two proposed solutions, DDS achieves slightly lower delay and energy costs than deep RL scheduling.

Djemai et al. [104] examine a network with multiple FNs and cloud nodes. MDs receive (from sensors and actuators) tasks modeled as directed graphs, and each node of these graphs represents a computational task that can be processed locally or in the fog or cloud nodes. The authors propose a solution (MH1) based on discrete PSO and compare it with six different baseline solutions including four simple ones like “cloud only” and two complex ones: binary PSO and the one from [64]. While the optimization problem aims to minimize energy consumption increased by violations of delay constraints, these values are only plotted separately. The proposed discrete PSO achieves lower energy costs and delay violations than the “simple” solutions. Comparison with binary PSO and the solution from [64] is less clear. Depending on the size of tasks, it can achieve both higher and lower energy consumption and delay violations.

Abbasi et al. [105] examine the same system as proposed by Deng et al. [97]. Unlike [97], they do not clearly state their objective function. They also miss some of the constraints used in [97] e.g., those requiring that offloaded tasks are processed. Still, their solution (MH4) to the problem is drastically different—they use NSGAII. They do not compare their solution against baseline solutions or those found in other works. Instead, they show results for three scenarios of possible workload allocation: only to FNs, only to clouds, and both to FNs and the cloud. There does not appear to be a clear gain from utilizing nodes in both tiers, as only FNs have the lowest delay and only clouds have the lowest energy consumption with both FNs and clouds achieving intermediate results.

He et al. [78] examine a network with multiple MDs and one or more FNs. MDs can offload their tasks or process them locally. However, the focus of optimization is on a single MD user whose offloaded tasks can be detected by adversaries. Scenarios with both colluding (C18) and non-colluding (C19) adversaries are examined. Similar solutions based on Lyapunov optimization are used for both scenarios to minimize energy consumption while preventing the detection of “feature” tasks. Baseline solutions include privacy-ignoring solutions and “naive” solutions that prohibit the offloading of “feature” tasks. Results show the performance gain (lower energy cost) of the proposed solution when compared with the naive one, as well as the price of privacy (higher cost) when compared with the privacy-ignoring one. They also show that a higher number of FNs to which tasks can be offloaded decreases costs for all solutions.

Nath and Wu [80] examine a network with multiple MDs, one or more FNs, and a cloud. MDs can offload their tasks or process them locally, and FNs can cache tasks that they process or fetch them from the cloud or other FNs. They propose two solutions based on Deep Deterministic Policy Gradient (DDPG): one for a network with a single FN (ML4) and a decentralized one for multiple cooperating FNs (ML5). For a network with a single FN, they compare the average cost (weighted sum of delay, energy, and fetching) with a total of eight baseline solutions. For all input parameters, their proposed solution achieves the lowest cost. They also compared their solution with a non-cooperative one and a centralized cooperative one for a network with multiple FNs. Understandably, their solution has higher costs than the centralized one and significantly lower than the non-cooperative one.

Roy et al. [106] examine a network with multiple MDs, multiple FNs divided into two hierarchical tiers (dew and edge), and a central cloud. Each task (called a subservice) can be offloaded to one of the FNs or cloud. The proposed solution (MH5) based on a GA and adaptive PSO is compared with solutions proposed in other works: one based on self-adaptive PSO [168], one based on binary PSO [170], one based on GA [105] (MH4.), and one from [171]. The results of simulations show that the proposed solution achieves a higher fitness (chosen objective to be maximized) than the baseline solutions. It also achieves both lower energy consumption and delay (i.e., parameters used to calculate fitness).

Zhang et al. [111] examine a network with multiple MDs and a single FN consisting of multiple computational servers of two different types. MDs can offload workload through RRHs or process it locally. The objective is to minimize the energy consumption divided by the total size of the processed workload (the authors call it the number of tasks rather than the size, but it is given in bits, and the whole model follows that of computational load rather than discrete tasks—see Section 3.3). The proposed multi-step solution (MA5) utilizes Lyapunov optimization to split the problem into four sequentially solved subproblems. The results achieved by the proposed solution are compared with those achieved by the following baseline solutions: no offloading, random, exhaustive search, and one where all steps follow the one from the proposed solution, but the two types of FN servers cannot share computation resources. The results show that the proposed solution achieves close-to-optimal energy efficiency (when compared with an exhaustive search) and significantly better efficiency than no offloading or random offloading. The difference resulting from different FN servers not being able to cooperate has no impact when there are lower arriving workloads and a small impact on energy efficiency as well as a significant impact on average delay when there are higher workloads.

Vakilian et al. [91,92,93] examine a network with multiple FNs and a cloud sharing workload offloaded from MDs. The differences between the models and optimization problems of these three works are relatively small. A solution based on Splitting Conic Solver (SCS) (C17) is used in [91]. Metaheuristics are chosen in the other works, i.e., Artificial Bee Colony (ABC) in [92] (MH6), and the cuckoo algorithm in [93] (MH7). ABC is shown to converge to optimal values found by SCS. Meanwhile, the cuckoo algorithm is shown to have lower energy consumption and delay than the solution proposed in [178]. All three works show that an increased number of cooperating FNs decreases the costs (weighted sum of energy consumption and delay) for a given workload.

Abdel-Basset et al. [94] examine a network with a single FN that allocates offloaded tasks to VMs. While the authors do not clearly state their optimization problem, we assume that their objective is to minimize fitness—a weighted sum of energy consumption and makespan (highest delay among VMs). The authors propose a solution based on MPA adapted to solve the discrete problem of task allocation. In total, they propose three versions: MPA (MH8); modified MPA (MH9), which changes the process of updating positions, and improved modified MPA (MH10), which adds ranking-based reinitialization and mutation. The proposed algorithms are compared with other metaheuristics: the sine cosine algorithm, the whale optimization algorithm, the salp swarm algorithm, the equilibrium optimizer algorithm, and the GA. The results show that improved modified MPA achieves the best results (lowest fitness, energy consumption, makespan, and CO_2_ emissions) for all studied scenarios. The second best is GA, better than the proposed modified MPA and MPA, which in turn perform better than the rest of the tested metaheuristics.

Bai and Qian [81] examine a network with multiple MDs and multiple FNs. MDs can offload their tasks to the FNs or a cloud or process them locally. The problem of the allocation of tasks to nodes as well as channel and computing resources to tasks is solved using the deep RL-based A2C algorithm (ML6). Random assignment is chosen as a baseline solution. The proposed A2C algorithm at the beginning of the simulation achieves a similar cost (weighted sum of delay and MD energy) to the random assignment, but with more episodes, its cost drops to less than 20% of that of the random assignment. They also show that the cost decreases with a lower number of MDs and a higher number of FNs, but there are diminishing returns after 20 FNs. The plot that shows these relations is inconsistent with its description in the text—there are data points that do not correspond to the described values, and there is an unexplained dip for 40 MDs.

Ghanavati et al. [113] examine a network with a broker, multiple FNs, and a cloud. The broker (gateway or AP) receives jobs (sets of tasks) from MDs and offloads them to FNs or a cloud. They propose an Ant Mating Optimization (AMO) algorithm (MH11) to solve the problem of minimizing the cost—the weighted sum of energy spent by FNs and the total delays for each job. They choose the following metaheuristics used in other works as a comparison: the bee life algorithm [172], the GA [173], and the PSO utilizing fuzzy logic [174]. The results show that the proposed solution achieves lower costs than the ones based on other metaheuristics. Its improvement in reducing delays is more significant than in reducing energy consumption. The bee life algorithm achieves the second-best results.

Sun and Chen [95] examine a network with multiple FNs and MDs, each MD connected to a single FN. An MD can offload a task to an FN by paying it a certain cost decided by the FN. The optimization problem for each FN is to maximize its utility, i.e., the price paid by MDs for offloading tasks subtracting various costs, which include energy. The baseline solutions include one that is Contract-based under Symmetric information (CS), one that is based on a Stackelberg game, and one that is based on linear pricing. The CS one achieves the highest utility for an FN. As it has all the information about offloading MDs, it can extract maximum revenue from them so that their utilities are zero. It can be seen as an upper bound. The proposed solution (MA12) has, therefore, lower utility than CS but higher than the other baseline solutions. It also achieves the highest utility of MDs.

Daghayeghi and Nickray [117] examine a network with multiple MDs-offloading tasks to FNs and cloud DCs through scheduler nodes. The goal is to optimize both energy consumption and delay. The proposed solution (MH12) is based on Strength Pareto Evolutionary Algorithm II (SPEA-II) and utilizes differential evolution to enhance the exploration of the search space. They compare the results of their algorithm against a total of five different baseline solutions proposed in other works, including the original SPEA-II from [175]. The authors perform parameter sweeps (size of tasks, task arrival rate) and provide detailed results in the form of multiple plots and tables. The results show that the proposed solution consistently achieves the lowest costs with the original SPEA-II performing second best. Interestingly, the proposed solution chooses to process a higher percentage of tasks in the clouds (36%) compared with baseline solutions (20–27%).

#### 5.4.3. Energy Only as a Constraint

Mao et al. [71] examine a network with a single MD capable of EH and an FN. The optimization problem is to minimize the average (over time) delay penalized by the dropped task and constrained by the battery charge level of the MD. In the proposed solution (MA2), the authors utilize Lyapunov optimization and find the optimal values for each decision variable step-by-step. They compare their method with three baseline solutions: no offloading, all offloading, and dynamic offloading, which chooses whether to offload based on what decision incurs lower delay. For all the studied scenarios, the proposed solution achieves the lowest cost, and the dynamic offloading achieves the second lowest. All offloading achieves the lowest average delay for most scenarios. However, it is compensated by a higher dropped task percentage—up to 50% for a scenario with a large distance (80 m) between the MD and the FN caused by lower channel gain.

Chen et al. [88] examine a network with multiple FNs that share the workload offloaded by the MDs. The problem is to minimize delay over time with constraints on energy over time, as well as per time slot energy and delay (see the definition of “over time” in Section 5.3). The authors propose a centralized optimization algorithm (C16) using Lyapunov optimization and a decentralized one (G1) reaching a Nash equilibrium through a best-response algorithm. The chosen baseline solutions are: one without FN–FN offloading, one ignoring the overtime energy constraint, and one transforming the overtime energy constraint into one that is stricter per time slot. The results show that both centralized and decentralized algorithms can satisfy overtime energy constraints. They have a lower average delay than the baseline solutions except for the energy-ignoring one. The performance gain of the centralized solution in relation to the decentralized one is clearly presented. It grows with higher variation in task arrival rate.

Wang and Chen [107] examine a network with multiple MDs offloading tasks to a single FN or processing them locally. The goal of minimizing the total delay while satisfying the delay and energy constraints of each task is solved using Hybrid Genetic Simulated Annealing (HGSA) (MH3)—a combination of GA and simulated annealing. The baseline solutions include no offloading, full offloading, and a version of HGSA used in [169]. The results show that the proposed solution achieves lower total delay than simple no-offloading and full offloading approaches. However, it has slightly higher total delay than the solution from [169], and the difference between the two increases with an increasing number of MDs. On the other hand, the proposed solution has lower total energy consumption than the one from [169]. Still, it is the delay, not the energy, that is the optimization objective.

Bian et al. [83,84] examine a system with multiple MDs and a single FN. The network performs distributed training, and the FN dynamically chooses MDs for each round of training. Their proposed solution (MA7) combines Lyapunov optimization with Upper Confidence Bound (UCB)-based graphical bandit learning. They compare their solution with baseline solutions: random MD selection and the “classical” UCB-based graphical bandit algorithm. Moreover, they do propose three slightly modified versions of their solution: one that does not utilize the graph information, one that ignores the energy constraint, and one that ignores the fairness constraint (MD minimum selection rate). All variations of the proposed solution achieve lower average latency than baseline solutions with the fairness-ignoring one having the lowest average latency and energy consumption by a large margin. However, it expectedly fails to reach the required fairness threshold. The solution without the graph information produces only slightly inferior results to the main one. The authors also study the effects of multiple network parameters and weights used by the solution on results such as average latency and testing accuracy.

### 5.5. Section Summary

**Figure 6:**
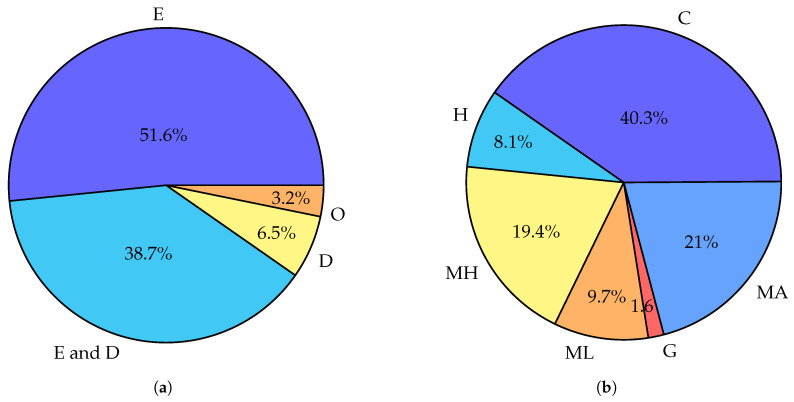
Chart summarizing solutions examined in Section 5. Objective function components: (E)nergy, (D)elay, (O)ther. Optimization method families: (C)onvex, (H)euristic, (M)eta(H)euristic, (M)achine (L)earning, (G)ame theory, (M)ixed (A)pproach. (**a**) Distribution of objective functions. (**b**) Distribution of optimization methods.

Many works have been published on the optimization of energy-efficient fog networks, which is still a timely topic. The examined works often provide multiple optimization problems and solutions to these problems. In these problems, energy consumption is used as either an optimization objective, a constraint, or both. Over half (32 out of 62) of the solutions use energy consumption as the objective function as shown in Figure 6. The objective function often includes summing the energy costs over multiple devices, sometimes with unequal weights as described in detail in the *Objective function* column of Table 5.

We find that most proposed solutions (25, 40.3% of the total) belong to the family of convex optimization, as presented in Figure 6b. There is also a significant share of algorithms (12, 19.4%) based on metaheuristics, all of which are nature-inspired. Another large portion (13, 21%) uses approaches from different optimization families to solve corresponding problems. Still, solutions from within the same family can vary greatly.

There is no clear trend as to which optimization algorithms are becoming more popular among researchers. There is also no easy answer to the question of which algorithms produce the best results. Still, based on the survey we have conducted, we provide general recommendations below:

1. Use the well-tested convex optimization methods if the problem you are examining can be solved with them.
2. Otherwise, test the performance of the existing metaheuristics or Machine Learning (ML) algorithms on your problem. Adjusing the method to better suit your particular problem is advisable.
3. As shown in Table 5, it can be beneficial to split the problem into smaller subproblems where each subproblem can be solved with a different method.

Some authors choose algorithms proposed in other research works as baseline solutions. In the vast majority of cases (except for when [75] compares itself with [70], and to a lesser extent when [107] compares itself with [169]) the proposed solutions show improvement over the cited ones. Still, most works use trivial baseline solutions such as no offloading or random assignment.

## 6. Discussion and Trends

We look at areas related to fog networks as an outcome of the survey presented in the previous sections. First, we discuss technologies applied to fog networks to reduce their energy consumption. Second, we tackle the topic of security and privacy. Third, the economic aspects of fog are discussed. Then, we look at the utilization of fog in smart agriculture. Finally, we discuss future research directions.

### 6.1. Fog Technologies

The main technology used for reducing energy consumption in fog networks is DVFS [153]. Depending on the requirements of computation tasks, either voltage or CPU frequency is adjusted in order to save energy. Furthermore, various variations of DVFS are available [179]. It is possible to dynamically scale only frequency or only voltage; however, as discussed in Section 3.5.1, operating at higher frequency requires appropriately higher voltage [152]. Furthermore, Near-Threshold Technology (NTV) targets the operation of a circuit at such low voltage levels that it may even result in computation errors. The errors are (by design) dynamically captured and addressed. The following of surveyed works utilize DVFS: [71,72,90,97,107,114,115].

Furthermore, the usage of System-on-Chip (SoC) architecture can reduce the power consumption of devices [180]. The SoC allows the grouping of various components such as CPU, memory, and various interfaces on a single chip. This in turn allows the joint optimization of circuit power consumption. Some particularly power-hungry components, such as Graphics Processing Units (GPUs), are areas for optimization themselves. On the contrary, SDN enables the dynamic management of devices installed in the cloud tier. Devices located in the core of the network used to be unavailable for dynamic management. This is currently changing thanks to SDN, which exposes devices to security threats though.

Energy consumption can be divided into the consumption of renewable and non-renewable (brown) energy. Subsequently, the reduction in brown energy consumption is more important than the reduction in renewable energy consumption. Energy harvesting, as well as the generation and usage of renewable energy, is beneficial here [87]. Energy harvesting is suitable for the things tier [50], while renewable energy (distributed via the electricity grid or generated locally) is suitable for devices located in the fog and cloud tiers. The availability of renewable resources is of high importance when designing or extending the network. This is related to the availability of energy sources such as wind turbines or solar panels. Energy that can be saved for cooling when placing DCs in cold locations of the globe is also relevant. Regarding existing networks, the usage of renewable energy requires changes in the network infrastructure, which usually entail disposal (energy) costs [181].

ML can be used to dynamically manage the network with the aim of energy reduction. Online learning is used in [87] to decide which tasks are computed in the fog and which are offloaded to the cloud, which in turn is autoscaled (dynamic computation and decisions on how many servers are active) [87,182].

Finally, a combination of various technologies can be used. E.g., DVFS can be used to minimize energy consumption on an SoC architecture. Renewable energy can be used across all the tiers in devices using SDN. ML can be applied to parts of the network, to single tiers, or even to single devices.

**Figure 7:**
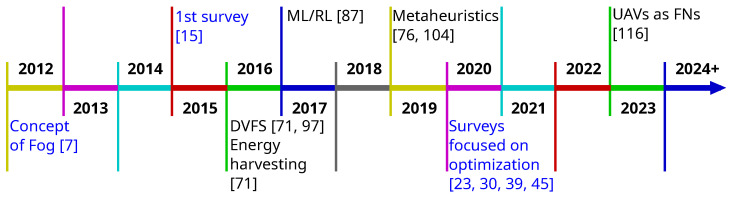
Timeline of key technologies (black) and general trends (blue) in fog research based on surveyed work [7,15,23,30,39,45,71,76,87,97,104,116].

Figure 7 shows a timeline of trends in fog research with key technologies outlined. Each topic represents the earliest use of said technology/algorithm in surveyed works and is accompanied by the respective reference. The concept of fog computing was proposed in 2012 [7] and after 3 years, the first survey work was published. In 2016, works on offloading in fog started including technologies such as DVFS and EH [71,97]. Then, we see the first of many works utilizing RL to solve optimization problems in fog [87]. Solutions based on nature-inspired metaheuristics started appearing in 2019 [76,104]. An increase in the number of works optimizing fog followed by surveys focused on resource allocation and optimization [27,30,31,33]. The utilization of UAVs as FNs is a relatively recent (2023) development [116].

### 6.2. Model-Based ML for Approaching Energy-Optimum

As discussed in the previous section, the optimization of energy efficiency or the minimization of energy consumption in a fog network requires knowledge of many parameters, characteristics, and statistics across multiple OSI-model layers and computing nodes. This knowledge may not be available to the extent that it is useful in theoretical optimization. On the other hand, utilizing data-based approaches, such as ML, may be extremely computationally complex and require huge learning datasets. Thus, a model-based ML [183] for the fog energy efficiency emerges as an approach that utilizes network structure (architecture) and traffic models to learn the communication and computing task classification and the place of their execution to satisfy the users’ requirements and constraints and minimize the energy consumption in the network.

The idea of the model-based ML is to integrate Artificial Intelligence (AI) techniques with traditional mathematical and numerical approaches. The combination of these two factors is crucial because, although AI/ML techniques are highly effective, they also greatly benefit from domain knowledge in the form of physical models. Applying AI techniques can be challenging because the effective integration of our understanding of physical processes into learning algorithms may be non-trivial. It has recently been demonstrated that traditional AI/ML techniques in networks are not scalable with network size because they fail to take into consideration the unique characteristics of the large-scale problem that needs to be solved.

Customizing and accounting for the unique properties of the fog networks through model-based ML should ensure scalability, generalization, reliability, and user trust [183]. Additionally, this approach enables technically robust and possibly explainable-by-design ML solutions. It should be further studied for application in energy-aware fog networks.

### 6.3. Security and Privacy

Telecommunication and cloud/edge/fog service providers must adhere to the existing regulations and standards unless new ones are created due to the development of new network functions. It is crucial to emphasize that service-based architectures and software-based virtualized networks are susceptible to hacker attacks. Issues that need to be addressed and included in the fog network functionalities include cybersecurity standards, the zero-trust concept, strict network monitoring and security procedures, security testing for software, hardware and user equipment, anomaly detection, threat protection, and data privacy [13,14,57,58,59,60].

The relationship between energy efficiency and security in the fog is outlined below:

- Resource management security: Fog computing relies on distributed resources that are geographically dispersed, including devices and DCs. Ensuring the security of these resources is essential for maintaining overall system efficiency. By implementing robust security measures such as access control, encryption, and authentication, the energy efficiency of fog infrastructure can be optimized without compromising security.
- Task classification security: Task execution in the fog architectures is based on task classification (e.g., with respect to required complexity, latency, reliability, bandwidth, etc.). Examples of such sensitive tasks include mission-critical tasks that require ultra-low latency and ultra-high reliability. The classification criteria may change, but considering the multi-class classification with defined sensitivity levels (from non-critical tasks to highly critical ones), any miss-classification may cause a fatal error in task execution, especially when the classes are close to the classifier. Moreover, it may translate to energy overspending if serving the task with inappropriate resources.
- Threat mitigation: In comparison to conventional cloud systems, energy-efficient fog devices could have less computational power and resources. Consequently, it is critical to recognize potential security risks and weaknesses that may compromise total energy efficiency. Energy resources can be preserved and used more effectively without being consumed by malicious activity by quickly identifying and addressing threats.
- Data privacy and integrity: Fog computing involves processing and storing data at the network edge, closer to the data source. This raises issues with data integrity and privacy. It is imperative to guarantee that the information conveyed, handled, and retained within the fog infrastructure is shielded from unwanted access, manipulation, or corruption. Energy-efficient fog systems may be trusted with critical data by putting strong security measures in place, which increase efficiency and dependability.
- Security-energy costs: Security procedures, threat detection, and mitigation algorithms come at an energy cost. At the same time, these algorithms may prevent the devices and network from energy overspending (e.g., by unnecessary retransmissions or network access attempts). Thus, it is necessary to thoroughly evaluate and balance the energy cost and benefit of security measures.

In summary, energy efficiency and fog computing security are closely linked. The overall effectiveness of fog infrastructure can be preserved or increased while upholding a secure environment by protecting resources, reducing risks, and guaranteeing data privacy and integrity.

### 6.4. Fog Economics

Economic factors of the fog have been the focus of the research area called *Fogonomics* [184]. Fogonomics affects the design of fog architectures, the economy of resource sharing, interactions between consumers and service providers, and resource pricing. The following challenges arise from the heterogeneity inherent in fog architectures: (i) coexisting heterogeneous networks, which necessitates selecting multiple network providers and interfaces; (ii) scaling computing needs and selecting the computing location by a range of end devices; and (iii) compliance with the required performance metrics of heterogeneous services offered [185].

With respect to the pricing of network communication resources, current mobile Internet Service Providers (ISPs) typically provide capped and usage-based data plans that demand a base fee for a finite number of data and deteriorate QoS or impose steep overage costs beyond this amount. A fog network can mitigate increasing network congestion by dynamically pooling resources and performing computations on local devices, which in turn reduces the amount of data that must be transferred and thereby lowers the cost of Internet access, including energy costs. Nevertheless, determining the right pricing for different QoS requirements is a difficult research problem because the devices have multiple options for network connectivity with different network interfaces.

Regarding computing resources, the majority of payments are made up of CPU, memory, and storage access costs. Dedicated instances with preset configurations or serverless services can be used to carry out the computational activities upon request. The use of resources on different devices by fog apps will result in various nonfinancial and financial expenses, the latter having a direct relation to the energy spent on computations. In order to select the optimal application configuration in terms of both cost and performance, cost optimization services are required.

One can designate specific devices to fulfill different functions in a heterogeneous fog scenario. When determining communication and computing resource pricing, this flexibility, as well as the trade-offs between the relevant QoS measures that are offered (such as energy efficiency vs. latency vs. dependability and security) should be taken into account. [184] contains case studies on resource pricing in these kinds of scenarios.

Pricing strategies that shape and respond to user demand and draw into a higher market share are needed for fog resources, especially energy. For these to guarantee the reliability of nodes providing resources and sharing-incentive mechanisms, surge pricing might be necessary. These could be volume-discount pricing, pay-per-use pricing, auction-based pricing, or taxation. Such pricing methods have the potential to alter demand structure in a way that reduces energy costs, the related carbon footprint, and traffic congestion while also having a major positive impact on customer satisfaction. They can increase revenue for service companies when properly built.

Recent progress in the architecture of fog networks, the optimization and management of communication, and computing resources in terms of energy efficiency, as well as the economics of their sharing, allows us to consider fog networks as a promising direction for reducing costs and developing telecommunications business.

An interesting direction that telecommunications companies can take is that of implementing a sharing economy model. This has been listed as a high-impact driver for the development of the telco industry [186]. Part of the sharing economy is called uberization, the process of creating a socio-economic system built around the sharing of resources [187]. In an uberized economy, services can be offered on demand between a customer and a supplier, who is often a freelancer [185]. Areas of telco business that are particularly suitable for resource sharing are infrastructure and spectrum [185].

There are examples of research works that test the feasibility of uberization in networks [188,189]. Song et al. [188] consider sharing the same resource blocks between two users in a Non-Orthogonal Multiple Access (NOMA) network. They develop a pricing scheme where one of the MD users can be seen as the driver and the other takes the role of a passenger. This model includes costs and revenues related to transmission power. Finally, their results show that utilizing this ride-sharing concept can allow for higher transmission rates, and it is especially well suited for NOMA networks. Meanwhile, Gall et al. [189] examine a relay-assisted D2D network in an urban environment. They propose a cost model for the deployment of such relays. It allows them to propose an uberization strategy in which an operator can set up a network with only D2D transmission. The results show that the number of users of such a network increases in a sigmoid-like pattern: first, there are no users before the commercial launch (1 year during which the network is still under deployment); then, there is a period of accelerating growth; and finally, there is slow growth and stabilization. The authors of [189] claim that such a deployment reaches Return on Investment (ROI) in 43 months.

All in all, while the works [188,189] showcase the potential of uberization, the networks they consider are not based on fog, and energy optimization is not their focus. To the best of our knowledge, there have not been any works focusing on energy aspect of uberized fog networks.

### 6.5. Future Research Directions

Future trends in fog communication and computing will focus on increasing broadly understood efficiency, enhancing security, and enabling more advanced applications. We believe that the following directions of research and future fog development will translate into its energy efficiency:

- Fog orchestration and management: As fog networks continue to grow, the efficient management and orchestration of resources will become crucial. Future research shall focus on developing advanced fog management frameworks to automate resource allocation, load balancing, and service provisioning, as well as on the energy cost to be paid for such management algorithms.
- AI at the edge: Fog computing combined with AI will enable intelligent decision-making at the edge of the network, reducing latency and improving real-time processing capabilities. However, the AI/ML algorithms themselves can be extremely computationally complex and thus can be highly energy-consuming. A trade-off between the energy consumption induced by these algorithms and the energy savings they offer has to be examined.
- Security of fog computing: Securing fog nodes and their communication will become essential. The implementation of robust security mechanisms, including encryption, authentication, anomaly and intrusion detection, continuous network monitoring, and methods counteracting cyberattacks will inevitably be energy-consuming. The cost of security (also in terms of energy consumption) needs to be assessed.
- Industry-specific fog applications: Different industries will leverage fog communication and computing for specific applications. This includes smart cities, autonomous vehicles, healthcare, industrial automation, and more. The tailored fog solutions designed to address the unique requirements of each industry will have a different impact on their energy consumption. Cost-benefit analysis for these specific application scenarios is another direction of research to be conducted.

A particular field where industry-specific fog applications have room to grow is smart agriculture. A survey [51] focused on the utilization of cloud/fog/edge computing in smart agriculture cites only 11 works examining edge and/or fog. On the other hand, Alwis et al. do not mention fog/edge at all in their survey [190] from 2022 with 80 references focused on processing data gathered by agricultural sensors. Still, we believe that the decentralized resources provided by fog networks could be well utilized by vast smart farms with numerous IoT sensors. This appears to be a promising (and underexplored) research direction.

Other trends and research directions include integration with the (co)existing cloud environments, 5G and future 6G systems, and any kind of distributed services. Overall, fog communication and computing will continue to advance, facilitating less energy-expensive, faster, more secure, and intelligent processing at the network edge. These trends will have a profound impact on a wide range of industries.

## 7. Conclusions

A plethora of works surveying fog networks have been published in recent years with varying degrees of focus on topics related to energy consumption and efficiency. We show with the extensive Table 1 how our work differs from these surveys. To the best of our knowledge, we are the first ones to provide a survey of energy-efficient fog network scenarios. We highlight the similarities and differences in how aspects of fog networking such as traffic, communication, and computation are parameterized. Our survey of energy-optimization methods in the fog is the most comprehensive to date.

Our work is focused on the energy consumption of networks utilizing fog, but, as the fog is meant to complement the capabilities of the cloud, we span our survey over the entire network from the IoT tier to the cloud tier (Figure 3). We consider the energy consumed for computation and for communication. Furthermore, we also examine network models used in the related works through the lenses of another key metric—the delay (see Table 2). We find out that computational task offloading is the main application type in the surveyed works, while traffic is usually modeled as discrete, indivisible tasks (see Table 2 and Figure 4). Conversely, in our energy consumption modeling, the energy consumed for communication traffic (both wireless and wired) is included. Moreover, in our work, we consider multiple parameters impacting computation-related energy consumption, e.g., the cost per clock cycle, the idle/active power consumption, the number of active servers, and the clock frequency. A summary of nonlinear energy models using clock frequency is shown in Table 3. The key lesson learned from this part of our survey is that modeling the energy consumption in a fog network requires architectural, traffic, system, and component details to be included in order to obtain and claim energy-efficiency feasible solutions. These required details of energy-consumption modeling are recommended in Section 3.

Before looking at methods used for the reduction in energy consumption in the modeled networks, we look at the scenarios used in the surveyed works. Values of parameters used in these scenarios have a significant impact on the results of optimization methods in terms of energy consumption. The following categories of parameters are distinguished in Table 4: 1. tasks/workload, which describes the traffic; 2. MDs, 3. fog nodes, and 4. cloud and its computational capability; 5. MD-FN communication, 6. intra-FN communication, and 7. FN-cloud communication that describe the connections between the nodes and their transmission characteristics. The main conclusion of this part of our survey is that no set of parameters is used as a benchmark to compare different works and that unrealistic simplifications are made to model the telecommunication traffic, and thus, there is a need (which is our recommendation for future work) to parameterize the network based on real-world data.

Finally, we provide a survey of approaches used to optimize fog with respect to energy consumption (Table 5). The approaches are grouped according to their methodology (e.g., convex optimization or heuristic) and according to the role that energy consumption plays in the problem (being the sole objective, being one of a few objectives, or being a constraint). We examine each problem and solution for works that tackle multiple problems and/or provide multiple solutions. We consistently discuss the results presented in the surveyed works and identify the baseline solutions that the authors use for comparison. One important finding of this part of the survey is that there is a need for approaching energy optimization based on the knowledge of network models on one hand and real-world data on the other hand. Model-based ML can be a viable solution for utilizing network architecture and traffic models and reducing algorithmic complexity.

Finally, future research directions and areas that are pivotal for reducing the energy consumption of future fog networks are as follows: (i) fog technologies, (ii) model-based ML approach to the energy optimum, (iii) security and privacy, (iv) fog economics, and (v) the sharing economy and uberization in the fog.

## Figures and Tables

**Table 3:** Comparison of nonlinear models of power (P) and energy (E) consumption.

	Order	2nd Degree P	3rd Degree P/ 2nd Degree E	Other
Type				
Monomial		–	MDs: [71,72,78,80,81,85,101,107,111,116] FNs: [90,95,102,107,111,116]	Cloud: [102] (3rd degree E)
Polynomial		FNs: [97,105]	FNs: [101,103,115], Cloud: [97,105]	FNs: [114] (nth degree P)

**Table 4:** Parameterization of the examined scenarios.

Parameters	Description	Works and Values	# of Works
1. Tasks/Workload			
1.1 Task/sub-task data size [kB]	Constant	0.125 [71,78]; 0.5 [85]; {2.5, 5, 7.5, 10, 12.5} [117]; 65.5 [98]; {100, 1000} [103]; {100, 200, …, 1000} [72]; 1000 [74]; 5000 [70]; {0, 625, …, 6250} [101]; 20,000 [116]	10
	Random: uniform <min, max>	<0.034, 66.5> [100]; <12.5, 62.5> [107]; <10, 100> [110]; <62.5, 125> [76]; <100, 500> [73]; <250, 1250> [86]; <1000, 5000> [81,115]; <200, 6000>[75]; <1000, 10,000> [82,114]; <1250, 12,500> [108]; <70,000, 80,000> [80]	14
	Random: exponential (mean)	25 [88]	1
	Directly stated, no values given	[69,79]	2
	Unspecified	[68,77,95,104,113]	5
1.2 Sub-tasks	Number per task	{2, 3, 4, 5, 6} [108]; 3 [104]; 8 [68]; 5 [113]	4
1.3 Workload data rate [Mbit/s]	Constant	{1.5, 2.5} [111], {500, 2000. 5000} [112]	2
	Real-world based (mean)	7 [90]	1
1.4 Arithmetic intensity [cycles/B]	Constant	0.125 [98]; 8 [86]; 50 [116]; {62.5, 75, 87.5} [74]; 92.2 [111]; {2, 20, 200} [103]; 297 [90]; 330 [70,72]; <550, 1100> [69]; 1000 [110]; 2678 [117]; {1000, 2222, 5000} [112]; 5600 [78]; 5900 [71]; 6400 [85]	16
	Random: uniform <min, max>	(0, 10> [95]; <0.5, 50>, <0.5, 250> [114]; <62.5, 187.5> [73,75]; <0.5, 250> [115]; <100, 1000>, <200, 1100>, …, <1000, 1900> [82]	6
	Random: quotient of two uniform distributions <min, max>	<5.63, 7.86> [80]; <0.5, 50> [108]; <10, 300> [81]; <160, 2400> [107]; <800, 8000> [76]	5
	Random: other (mean)	1600 [88]	1
	Directly stated, no values given	[79,113]	2
	Unspecified	[68,77,100,101,104]	5
1.5 Arrival of tasks	One at a time	[71,76,88,95,98,100,103,108]	8
	In groups	[72,73,75,79,80,81,82,85,86,104,107,113,114,115,116,117]	16
	Unspecified	[68,69,70,74,77,78,101,110]	8
1.6 Arrival distribution	Constant	[71,73,80,85]	4
	Random: exponential (Poisson)	[74,76,88,100,103,108,114,115]	8
	Real-world-based	[79,90]	2
	Unspecified	[68,69,70,72,73,75,77,78,81,82,86,95,98,101,104,107,110,113,116,117]	20
2. Mobile Devices			
2.1 Number of MDs	1	[68,70,71,72,77,78,85]	7
	Few (2–19)	[69,74,75,76,80,81,82,86,95,104,107,110,111,115]	14
	Many (20–99)	[73,76,79,81,86,107,111,114,116,117]	10
	Hundreds (100–999)	[81,88,98,114]	4
	Thousands (1000+)	[98,100,112]	3
	Unspecified	[101,113]	2
	None	[90,103,108]	3
2.2 Computational cost [nJ/cycle]	Constant	0.1 [81]; 0.2 [77]; 1.0 [80]; {1.11, 1.14} [104]; 1.37 [82]; 2.08 [70]; {4.38, 6.78} [79]; $1\times 10^{8}$ [74]; (*) [76]	9
	Random: uniform <min, max>	(0, 0.2) [73,75]	2
	Depending on frequency	[71,72,78,85,101,107,111,116]	8
	Unspecified	[68,69,95]	3
	N/A—no computation by MDs	[86,88,98,100,110,112,113,114,115,117]	10
2.3 Computational capability [Gcycles/s]	Constant	{0.0016, 0.0018, 0.002} [74]; 0.4 [70]; 0.5 [82]; {0.1, 0.2, …, 1} [73]; {0.2, 0.4, 0.6, 0.8, 1} [76]; 1.0 [80,81]; {1.7, 2.7} [79]; {2.8, 4.5} [104]	9
	Random: uniform <min, max>	<0.5, 1.0> [76]	1
	Depending on frequency	<0.15, 0.6> [107]; <0.2, 0.8> [72]; <0, 1.5> [71,85]; <0, 1> [116]; [78,101,111]	8
	Unspecified	[68,69,75,77,95]	5
	N/A—no computation by MDs	[86,88,98,100,110,112,113,114,115,117]	10
2.4 Mobility	Yes	[68,80,101,104,115]	5
	No	[69,70,71,72,73,74,75,76,77,78,79,81,82,85,86,88,95,98,100,107,110,111,112,113,114,116,117]	27
3. Fog Nodes			
3.1 Number of FNs	1	[70,71,72,73,74,75,76,77,78,80,85,98,107,111,116]	15
	Few (2–9)	[69,72,79,81,82,95,98,103,104,110,116]	11
	Many (10–99)	[81,86,98,103,108,113,114,115,117]	9
	Hundreds (100–999)	[88,90,100]	3
	Unspecified	[101,112]	2
	None	[68]	1
3.2 Computational cost [nJ/cycle]	Constant	{1.65, 3.7, 5.2} [104]; 8.2 [88]; {32, 65.5} [112]; 80 [98]; (*) [117]	6
	Random: uniform <min, max>	<0.8, 1.0> [108]	1
	Depending on frequency	[90,95,101,103,107,111,114,115,116]	9
	Directly stated, no values given	[100,113]	2
	Unspecified	[110]	1
	No cost	[69,70,71,72,73,74,75,76,77,78,79,80,81,82,85,86]	16
3.3 Computational capability [Gcycles/s]	Constant	0.004 per task, 0.04 total [74]; 0.8 [70]; 1.26 [98]; {0.2, 0.4, … 2.4} [117]; {2, 2.2} [72]; {1.8, 4} [112]; 5 [79,80]; {1, 3, 5, 7, 10, 15, 20} [81]; {4, 8} [90]; 6 [73]; 10 [69,82]; {6.75, 13.5, 60} [104]; {10, 20, 30, 40, 50} [76]; 150 [85]	16
	Random: uniform <min, max>	<0.01, 0.015> [86]; <10, 50> [108]	2
	Depending on frequency	<0, 3> [116]; {2, 3} [103]; <1.6, 4.2> [114,115]; <0, 15> [107]; [95,101,111]	8
	Directly stated, no values given	[113]	1
	Infinite	[71,73,75]	3
	Unspecified	[77,78,88,100,110]	5
Parameters	Description	Works and values	# of works
4. Cloud			
4.1 Number of clouds	1	[68,72,74,79,81,82,90,95,98,103,104,108,110,114,115,116]	16
	Few (2–9)	[100,117]	2
	Unspecified	[112]	1
	None	[69,70,71,73,75,76,77,78,80,85,86,88,101,107,111,113]	15
4.2 Computational cost [nJ/cycle]	Constant	0.2 [108]; <0.33, 1.25> [115]; {0.1, 1, 2} [103]; <0.2, 2> [114]; 1.44 [104]; 80 [98]; 462 [112]; (*) [117]	9
	Unspecified	[100,110]	2
	No cost	[68,72,79,81,82,90,95,116,154]	9
4.3 Computational capability [Gcycles/s]	Constant	0.01 per task [74]; 1.5 per task [103,114,115]; {4, 5, 6} [117]; {4, 10} [72]; 10.64 [112]; 10 per task, 40 total [82]; 50 [79]; 120 [104]; 125 [98]; 200 [108];	12
	Infinite	[81,90]; total [74,103,114,115]	6
	Unspecified	[68,95,100,110,116]	5
5. MD-FN Communication			
5.1 Type of transmision	Wi-Fi	[68,82,110,114,115,117]	6
	Cellular—TDMA	[73]	1
	Cellular—OFDMA	[73,75,76,77,85,111,116]	7
	Cellular—general	[68,69,70,72,74,80,95,101,104,107]	10
	Unspecified	[71,78,79,81,88,98,100,113]	8
	N/A	[86,90,103,108]	4
5.2 Power consumption [W]	Directly stated	{0.01, 0.03, 0.04, 0.15} [68]; {0.1} [117]; {0.1, 0.2, 0.3, 0.4, 0.5} [76]; 0.9 [107]; 1.22 [113]; {1.18, 1.26} [72]; 2.51 [69]	7
	Indirectly stated	[70,71,73,74,75,77,78,80,81,85,88,115,116]	13
	Not included	[79,82,95,98,100,101,104,110,111,114]	10
5.3 Transmission cost [nJ/bit]	Directly stated	0.167 [117]; 2.5 [98]; 142 [79,82]; 407 [113]	5
	Directly stated, no values given	[101,104]	2
	Indirectly stated	[68,69,70,71,72,73,74,75,76,77,78,80,81,85,88,100,107,111,115,116]	19
	No cost	[95,110,114]	3
5.4 Transmission rate [Mbit/s]	Directly stated	2 [101]; {0.8, 1, 4, 6} [68]; 3.01 [113]; 30 [107]; {0.25, 1, 1000} [104]; {0, 6.5, 13, 18.5, 26, 39, 52, 58.5, 65} [115]; 72 [79]; 600 [110,117]; 1000 [100]	10
	Indirectly stated	[69,70,71,72,73,74,75,76,77,78,80,81,82,85,88,95,98,111,114,116]	19
5.5 Interference consideration	Intra-cell/Intra-FN	[74,77,80,95,114]	5
	Inter-cell/Inter-FN	[69,76,111]	3
	No	[68,70,71,72,73,75,78,79,81,82,85,88,98,100,101,104,107,110,113,115,116,117]	22
5.6 Choice of FN	MD always transmits to the same/closest FN	[69,70,71,73,74,75,76,77,78,79,80,85,88,95,98,100,101,104,107,110,111,113,114,117]	24
	MD can choose an FN	[68,72,81,82,115,116]	6
6. Inter-FN Communication			
6.1 Type of transmission	Wired	[74,79,88,103,104,108,110,114,115,117]	10
	Wireless	[76,86,90]	3
	Unspecified	[80,98,100,113]	4
	N/A	[68,69,70,71,72,73,75,77,78,81,82,85,95,101,107,111,116]	17
6.2 Power consumption [W]	Directly stated	0.25 [117]; 3.3 [103]; 82 [115]	3
	Indirectly stated	[86,90]	2
	Not included	[74,76,79,80,88,98,100,104,108,110,113,114]	12
6.3 Transmission cost [nJ/bit]	Directly stated	0.2 [108]; 0.25 [117]; 0.3 [103,114]; {4, 6} [115]	5
	Directly stated, no values given	[104]	1
	Indirectly stated	[86,90]	2
	No cost	[74,76,79,80,88,98,100,110,113]	9
6.4 Transmission rate [Mbit/s]	Directly stated	<5,10> [108]; 72 [79]; 100 [88]; 1000 [103,114,115,117]; 1500 [110]; 10,000 [104]	9
	Indirectly stated	[76,86]	2
	Infinite rate/negligible delay	[74,80,90,98,100,113]	6
7. FN-Cloud Communication			
7.1 Type of transmission	Wired	[72,81,82,90,95,98,100,103,104,108,110,114,115,117]	14
	Wireless	[68,116]; MD-Cloud [82]	3
	Unspecified	[74,79,80]	3
	N/A	[69,70,71,73,75,76,77,78,85,86,88,101,107,111,113]	15
7.2 Power consumption [W]	Directly stated	1.2 [117]; {5.5, 6570} [103]	2
	Not included	[68,72,74,79,80,81,82,90,95,98,100,104,108,110,114,115]	16
7.3 Transmission cost [nJ/bit]	Directly stated	0.3 [117]; 2.5 [98]; 8 [108]; 10 [114]; {6.38, 12.6, 18.7} [103]; 12.7 [115]; {278, 658} MD-Cloud [82]	7
	Directly stated, no values given	[104]	1
	Indirectly stated	[100]	1
	No cost	[68,72,74,79,80,81,82,90,95,110,116]	11
7.4 Transmission rate [Mbit/s]	Directly stated	{1, 2, …, 10} [82]; 72 [79]; 1000 [72,114]; 4000 [117]; 10,000 [100,103,104,115]; 40,000 [110]	10
	Indirectly stated	[74,81,98,108]; MD-Cloud [82]	5
	Infinite rate/negligible delay	[68,80,90,95,116]	5

## Data Availability

The original contributions presented in the study are included in the article, further inquiries can be directed to the corresponding author.

## Abbreviations

The following abbreviations are used in this manuscript:

A2C	Advantage actor–critic
ABC	Artificial Bee Colony
ADMM	Alternating Direction Method of Multipliers
AI	Artificial Intelligence
ALR	Adaptive Line Rate
AMO	Ant Mating Optimization
AP	Access Point
BB	Branch and Bound
BS	Base Station
CDN	Content Delivery Network
CMOS	Complementary Metal-Oxide Semiconductor
CS	Contract-based under Symmetric information
CPU	Central Processing Unit
D2D	Device-to-Device
DAG	Directed Acyclic Graph
DC	Data Center
DDS	Deep Dynamic Scheduling
DDPG	Deep Deterministic Policy Gradient
DL	DownLink
DVFS	Dynamic Voltage and Frequency Scaling
EH	Energy Harvesting
EON	Elastic Optical Network
FFBD	Feasibility Finding Benders Decomposition
FI	Fog Instance
FLOP	Floating Point Operation
FLOPS	Floating Point Operations per Second
FN	Fog Node
GA	Genetic Algorithm
GPU	Graphics Processing Unit
HGSA	Hybrid Genetic Simulated Annealing
ICT	Information and Communication Technology
IRS	Intelligent Reflective Surface
IoT	Internet of Things
IP	Internet Protocol
ISP	Internet Service Provider
KKT	Karush–Kuhn–Tucker
LNA	Low Noise Amplifier
LO	Local Oscillator
LP	Linear Programming
LTE	Long Term Evolution
LWS	Lone Wolf Search
MCC	Mobile Cloud Computing
MD	Mobile Device
MDP	Markov Decision Process
MEC	Mobile/Multi-Access Edge Computing
MILP	Mixed-Integer Linear Programming
MIMO	Multiple-Input and Multiple-Output
MINLP	Mixed-Integer Non-Linear Programming
ML	Machine Learning
MPA	Marine Predators Algorithm
N/A	Not Applicable
NEP	Nash Equilibrium Problem
NIC	Network Interface Card
NFV	Network Function Virtualization
NOMA	Non-Orthogonal Multiple Access
NSGAII	Non-dominated Sorting Genetic Algorithm II
NTV	Near-Threshold Technology
OFDMA	Orthogonal Frequency-Division Multiple Access
PA	Power Amplifier
PDS	Post-Decision State
PGN	Portable Game Notation
PON	Passive Optical Network
PSO	Particle Swarm Optimization
QoS	Quality of Service
RF	Radio Frequency
RL	Reinforcement Learning
ROI	Return on Investment
RRH	Remote Radio Head
SCA	Successive Convex Approximation
SCS	Splitting Conic Solver
SDN	Software Defined Network
SDR	SemiDefinite Relaxation
SID	Solution IDentifier
SoC	System-on-Chip
SPEA-II	Strength Pareto Evolutionary Algorithm II
TDMA	Time Division Multiple Access
UAV	Unmanned Aerial Vehicle
UCB	Upper Confidence Bound
UL	UpLink
VC	Virtual Cluster
VM	Virtual Machine
WDM	Wavelength Division Multiplexing
